# Single‐Atom Catalyst‐Integrated Porous Organic Polymers for High‐Performance Lithium‐Sulfur Batteries

**DOI:** 10.1002/smll.202503250

**Published:** 2025-06-16

**Authors:** Yun‐Sheng Ye, Mohamed Gamal Mohamed, Nai‐Hua Ye, Ting‐Yun Hung, Guan‐Yu Chen, Shi‐Hsin Lin, Meng‐Che Tsai, Bing‐Joe Hwang, Shiao‐Wei Kuo

**Affiliations:** ^1^ Department of Materials and Optoelectronic Science Center of Crystal Research National Sun Yat‐Sen University Kaohsiung 80424 Taiwan; ^2^ The Ministry of Education of Taiwan (the Sustainable Electrochemical Energy Development Center (SEED Center) from the Featured Areas Research Center Program National Taiwan University of Science and Technology Taipei 106335 Taiwan; ^3^ Chemistry Department Faculty of Science Assiut University Assiut 71515 Egypt; ^4^ Center of Crystal Research & Research Center for Physical Properties and Microstructure of Metals National Sun Yat‐sen University Kaohsiung 80424 Taiwan; ^5^ Department of Greenergy National University of Tainan Tainan 700301 Taiwan; ^6^ Department of Chemical Engineering National Taiwan University of Science and Technology Taipei 106335 Taiwan

**Keywords:** lithium sulfur battery, porous organic polymer, single atomic catalyst, tetrathiocine coordination

## Abstract

Lithium‐sulfur (Li‐S) batteries exhibit high energy density potential but suffer from lithium polysulfide (LPS) shuttling and sluggish conversion kinetics, hindering practical application. Here, a novel approach incorporating out‐of‐plane single‐atom catalysts (SACs) into a tetrathiocine‐linked porous organic polymer (POP) framework is introduced. This design enables precise spatial distribution of active metal sites, enhancing interactions with soluble LPSs. The out‐of‐plane configuration further supports unique coordination motifs, accelerating the transformation of soluble LPSs to solid phases and effectively mitigating the shuttle effect. The resultant Pt‐based SAC separator achieves outstanding catalytic efficiency, cycling stability, and capacity retention under high sulfur loading. The findings establish a foundational strategy that integrates advanced molecular design with electrochemical performance, offering a promising avenue for improving the practicality and efficiency of Li‐S battery technology.

## Graphical abstract

Out‐of‐plane single‐atom catalysts are developed for Li‐S batteries to improve redox kinetics and mitigate LPS shuttling. A thianthrene‐based POP with a tetrathiocine linker is synthesized to anchor Pt single‐atom catalysts with both in‐plane and out‐of‐plane coordination, enhancing LPS adsorption and conversion in Li‐S batteries. Pt‐S─POP/rGO‐modified separators suppress LPS shuttling, accelerate redox kinetics, and improve sulfur utilization. DFT confirms strong Pt–S interactions, lowering Gibbs free energy for efficient LPS conversion.

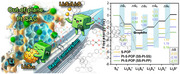

## 1 Introduction

**Scheme 1:**
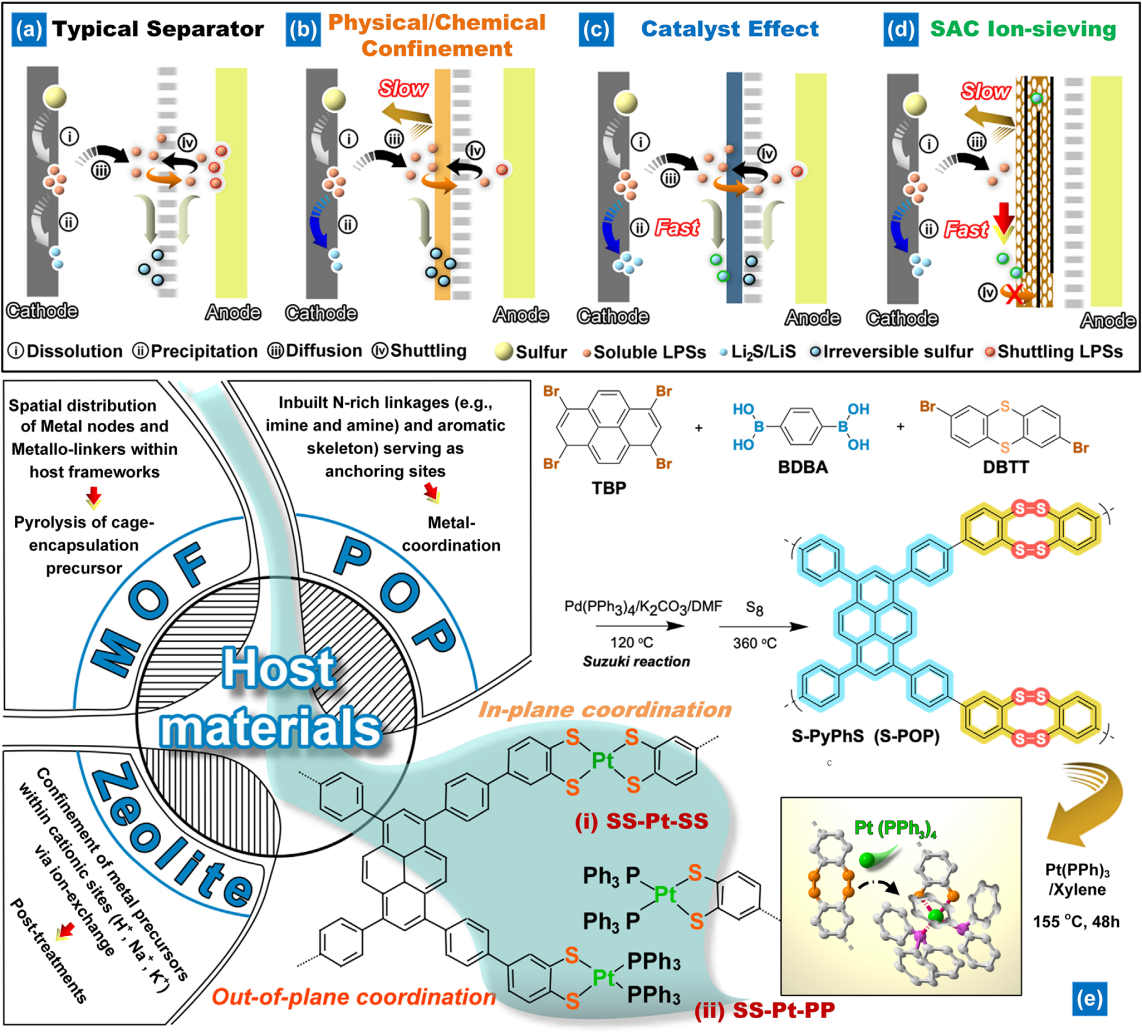
Approaches to deal with the problems of shuttling and sluggish conversion of LPSs include: a) using a standard separator, b) employing physical/chemical confinement, c) taking advantage of catalyst effects, and d) utilizing SAC ion‐sieving. The Li‐S battery operates in the following manner in a typical separator: 1) Elemental sulfur undergoes electron transfer and forms long‐chain LPSs (Li_2_S_n_) on the cathode side; 2) Some of the Li_2_S_n_ that are confined within a conductive substrate are further reduced to solid Li_2_S_2_/Li_2_S; 3) The Li_2_S_n_ that are not confined within the substrate are released and dissolved in the electrolyte, diffusing throughout it; 4) Some of the dissolved Li_2_S_n_ migrate to the Li metal side, while others are reduced to irreversible sulfur on or within the separator; the migration of Li_2_S_n_ leads to a continuous loss of active sulfur and the corrosion of Li anodes. By implementing physical/chemical confinement in the Li‐S battery, the diffusion of Li_2_S_n_ through the separator is hindered, but this can lead to the formation of irreversible sulfur within the trapped Li_2_S_n_. By leveraging the catalyst effect, the slow transformation of confined Li_2_S_n_ into solid Li_2_S_2_/Li_2_S within a conductive substrate is facilitated; however, the soluble LPS that has not been promptly converted and obstructed will still experience shuttling. In this study, we propose the use of SAC ion‐sieving as a fundamental solution to prevent the shuttle effect, which is achieved by impeding the diffusion of soluble Li_2_S_n_ and promoting the rapid deposition of Li_2_S_n_ into solid Li_2_S_2_/Li_2_S.

The lithium‐sulfur (Li‐S) battery has garnered attention as a promising energy storage device due to its low mass density, high theoretical energy density (1675 mAh g⁻¹), and energy density (≈2500 Wh kg⁻¹), along with its abundance, low cost, and environmental benefits.^[^ ^1^ ^]^ However, its practical application is hindered by low durability and poor cycle stability, primarily due to two issues: shuttling and sluggish polysulfide (LPS) conversion.^[^ ^2^ ^]^ The shuttling effect occurs when long‐chain, highly soluble LPSs (Li₂S_n_, *n* = 4–8) in the electrolyte cause severe corrosion of the Li metal anode during the charge/discharge process (**Scheme** **1**a), resulting in irreversible sulfur loss and increased internal resistance, which leads to diminished battery performance.

The solution to this issue is straightforward: stabilize the active material in the cathode and prevent the diffusion of soluble LPSs across the separator.^[^ ^1^, ^3^ ^]^ This can be achieved by integrating functional substrates that absorb the active material and/or LPSs within cathodes, interlayers, or modified separators (Scheme 1b).^[^ ^4^ ^]^ Additionally, incorporating a porous structure into these substrates helps to confine the active material to the cathode or act as a barrier, preventing LPS diffusion. These modifications significantly improve Li‐S cell performance by enhancing specific capacity and extending cycling life. While this method limits the shuttle effect, continuous LPS production during the charge/discharge cycle, if not intercepted promptly and fully, hinders the full benefits of an ideal sulfur battery. By accelerating LPS conversion between liquid LPSs and solid S/Li₂S₂/Li₂S, the amount of soluble LPSs in the electrolyte can be reduced, further boosting redox conversion kinetics for LPSs.^[^ ^5^ ^]^ Electrocatalysts, particularly metal‐derived ones, have been employed to catalyze the conversion of LPSs in Li‐S batteries.^[^ ^6^ ^]^ However, metal electrocatalysts with small specific surface areas may not offer significant catalytic effects or effectively impede LPS migration (Scheme 1c). Recently, single‐atom catalysts (SACs), with metal atoms dispersed across various matrices, have gained attention for their high intrinsic activity, selective catalytic properties, and low cost, offering substantial potential for Li‐S battery improvement.^[^ ^7^ ^]^

A key approach in SAC synthesis involves spatially confining individual metal atoms within molecular‐scale cages, such as zeolites, metal‐organic frameworks (MOFs), and porous organic polymers (POPs), to prevent atom migration.^[^ ^8^ ^]^ POPs, known for their large surface areas, structural tunability, and predictable synthesis, are ideal carriers for SACs in Li‐S batteries.^[^ ^9^ ^]^ To achieve uniform metal atom distribution and strong metal–support interactions (M‐N or M‐O bonds) in POPs, donor groups like bipyridine,^[^ ^10^ ^]^ salen,^[^ ^11^ ^]^ or porphyrin^[^ ^12^ ^]^ can be incorporated as ligands. When a single‐atom active center in POPs interacts with heteroatoms (O, Cl, S), out‐of‐plane coordination alters electronic properties, enhancing catalytic activity.^[^ ^10^, ^13^ ^]^ Despite progress, two main challenges remain: i) few host materials with suitable metal–support interactions can stabilize single metal atoms, and ii) the impact of SACs with out‐of‐plane coordination on the reversible LPS deposition and dissolution process is still unclear but significantly affects sulfur utilization and electrochemical performance.

In this work, we report the first synthesis of a novel porous organic polymer (POP) featuring active benzenediothiolate ligands designed to stabilize SACs via sulfurization of thianthrene‐based POP (C─S─C) (Scheme 1e). Pt‐based SACs (Pt‐SACs) are securely anchored to the tetrathiocine linkers (C─S─S─C) of sulfurized POP (S─POP) via Pt‐S bonds in a non‐planar coordination, optimizing electron distribution and d‐band positions within the Pt‐S catalysts. The resulting Pt‐coordinated POP (Pt‐S─POP) contains ≈6 wt.% Pt, which enhances long‐chain LPS conversion while its porous structure allows rapid Li⁺ ion transfer and easy access to LPSs. Leveraging the pyrene structure, Pt‐S─POP is hybridized with large‐area graphene oxide (LGO) to form an ion‐sieving layer on polyethylene (PE) using a simple blade coating method, eliminating the need for polymer binders. Following hydriodic acid and acetic acid treatments (HI‐AcOH), Pt‐S─POP/reduced graphene oxide (rGO)‐modified separators yield an ultrathin, lightweight coating layer (<150 nm, ≈0.04 mg cm⁻^2^) that significantly reduces LPS diffusion and accelerates liquid‐solid reactions for LPS conversion (Scheme 1d). Li‐S batteries incorporating Pt‐S─POP/rGO demonstrate exceptional cycling stability, maintaining consistent capacity over 2000 cycles at 1 C. These batteries also achieve a high initial areal capacity of 1.8 mAh cm⁻^2^ at 0.5 C, even with high sulfur loadings (>4 mg cm⁻^2^). This approach offers a fundamental strategy for mitigating the shuttle effect, marking a significant advancement in SAC development for Li‐S batteries.

## 2 Results and Discussion

### 2.1 Synthesis and Characterization

**Figure 1:**
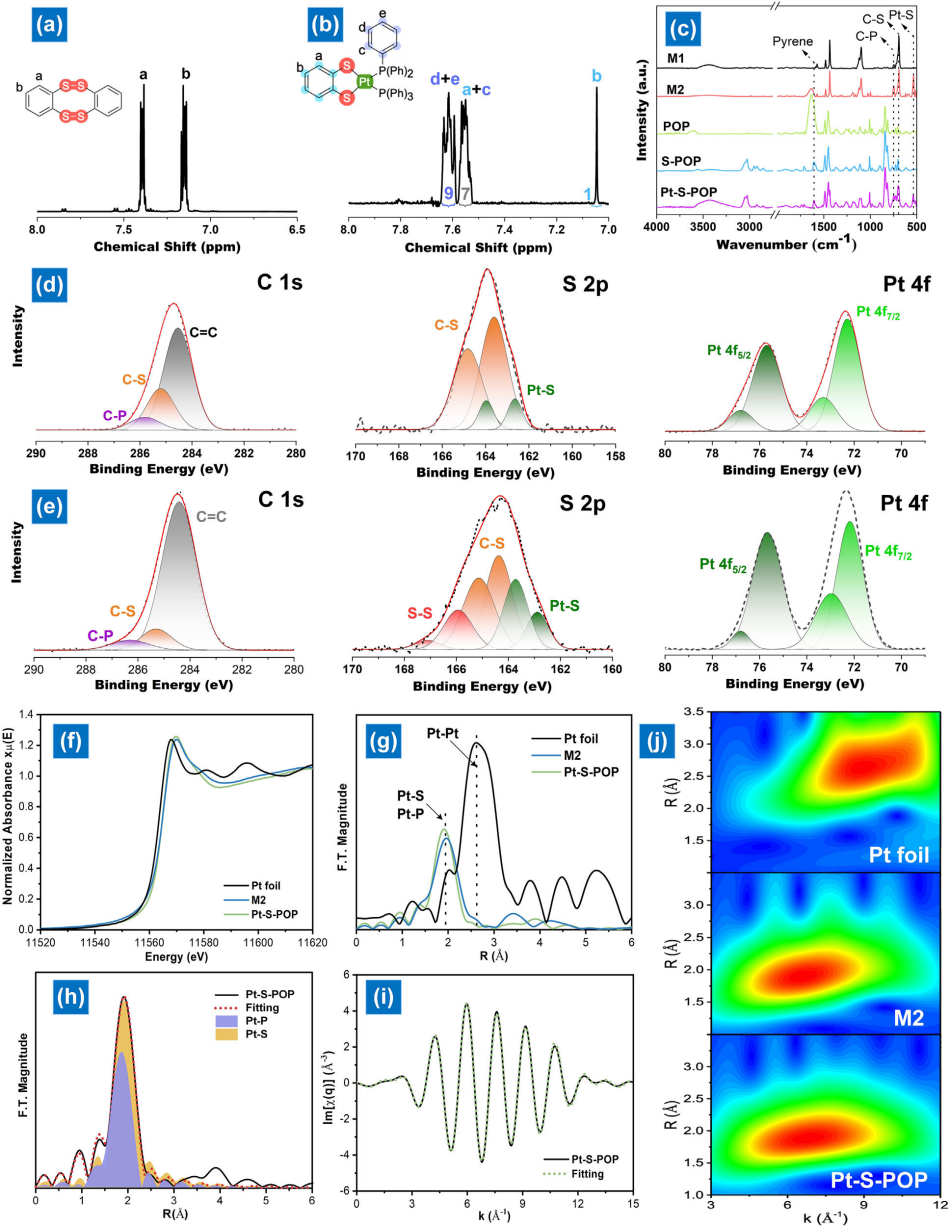
NMR spectra of a) M1 and b) M2; c) FTIR spectra of M1, M2, and POPs; High‐resolution XPS spectra of the C 1s, S 2p, and Pt 4f fitting for d) M2 and e) Pt‐S─POP. f) Pt L_3_‐edge X‐ray absorption near edge structure (XANES) spectra of Pt foil, M2, and Pt‐S─POP; g) FT‐EXAFS at the Pt L_3_‐edge; h) FT‐EXAFS fitting curves in the R space of Pt‐S─POP; i) XANES spectra in q space and fitting curves of Pt‐S─POP; j) Pt L_3_‐edge WT‐EXAFS contour plots of different samples.

A thianthrene‐linked POP was synthesized via Suzuki coupling, involving the reaction of tetrabromo pyrene (four connection points) with dithianthrene linkers (C─S─C, two connection points) using diboronic acid (Scheme 1). The thianthrene‐based POP was then mixed with sulfur in a sealed ampoule at an optimized temperature (see the Supporting Information for details) to convert C─S─C linkers into tetrathiocine ligands (C─S─S─C), following the method of Haldar and Kaskel et al.^[^ ^14^ ^]^ These ligands were subsequently coordinated with Pt using Pt(PPh₃)₄. In parallel, thianthrene was used to create model compounds for studying sulfurization and coordination in comparison to the thianthrene‐based POP (Scheme S1, Supporting Information). The powdered samples of the sulfurized model compound (M1) and the Pt‐coordinated model compound (M2) were thoroughly washed and purified via recrystallization. **Figure** **1**a shows the characteristic signals of aromatic protons in M1, detected at 7.15 and 7.39 ppm. Successful sulfurization of thianthrene was confirmed by mass spectrometry, which recorded a mass‐to‐charge ratio (*m*/*z*) of 280 g mol^−^¹, close to the theoretical value for M1 (Figure S1, Supporting Information). Additionally, a novel peak at 7.60–7.64 ppm indicates aromatic protons from the phosphine co‐ligand (PPh₃) (Figure 1b). The integral area of the benzenediothiolate ligand in M1 shows a 1:1 ratio with the phosphine co‐ligand, confirming the expected M2 complex.

The chemical integrity of the C─S─S─C linkage and the presence of coordinated Pt in POP are substantiated by a comparative analysis with a model molecule. The characteristic signals of the 535 and 749 cm^−1^ correspond to Pt‐S and C─P bonds in the infrared spectroscopy (IR) spectra for the Pt‐coordinated POP (Pt‐S─POP), and a similar signal position was noticed for the M2 (Figure 1c). Although the IR analysis faces difficulties in determining the conversion of C─S─C to C┐S─S─C bonds due to their similar electronegativity. However, the element analysis (EA) reveals that the sulfur content of 4.9 wt.% in POP was doubled to 10 wt.% after sulfurization, indicating the incorporation of additional sulfur atoms to form S─S bonds (Table S1, Supporting Information). By analyzing the X‐ray photoelectron spectroscopy (XPS) data from M1 and M2 (shown in Figure S2, Supporting Information and Figure 1d), we observed weak signals of 163.5 and 164.6 eV corresponding to organic disulfides in the S 2p measurement of the S─POP compound and the appearance of a Pt‐S signal at 162.6 and 164.0 eV following the coordination of Pt(PPh_3_)_4_. This is accompanied by shifting of the binding energy of S 2p_1/2_ and S 2p_3/2_ to a higher energy after the conversion to the hardly ionizable benzenediothiolate ligand. In addition, the presence of a peak at 285.8 eV in the C 1s spectrum and peaks at 72.3/73.3 eV (Pt 4f_7/2_) and 75.7/76.8 eV (Pt 4f_5/2_) in the Pt 4f spectrum indicate the coordination of Pt. When comparing the M2 based on XPS with the synthesized Pt‐S─POP (Figure 1e), it is observed that they exhibit similar characteristic signals of C 1s, S 2p, and Pt 4f, which are associated with the benzenediothiolate ligand, phosphine co‐ligand, and coordinated Pt. The presence of uncoordinated Pt tetrathiocine linkage is also identified by the signal of S─S bonds at 165.9 and 167.1 eV in the Pt‐S─POP. According to the ICP result, the amount of coordinated Pt in the Pt‐S─POP can be accurately analyzed as 6.0 wt.%, which value is similar to the calculated value of 6.9 wt.% determined by the char yield at 800 °C from the thermogravimetric analyzer (TGA) result (Table S1, Supporting Information).

**Figure 2:**
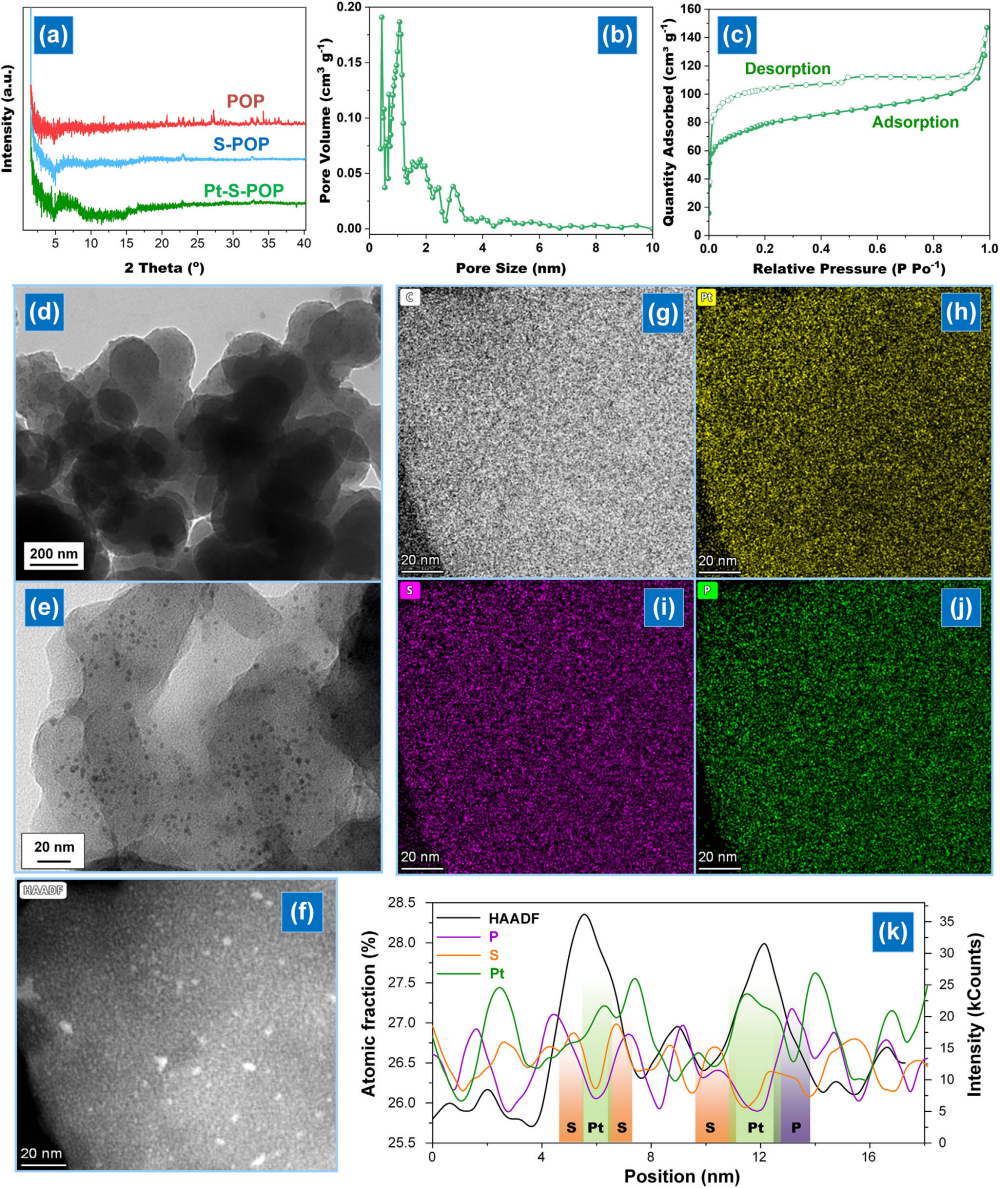
a) XRD pattern of POP, S─POP, and Pt‐S─POP; b) Pore width and cumulative pore volume distribution obtained through the BJH method from the desorption branch of the N_2_ isotherm at 77 K; c) N_2_ physisorption isotherm N_2_ of Pt‐S─POP recorded at 77 K; d,e) TEM and f) high‐angle annular dark‐field STEM images and g–j) STEM‐EDS elemental mapping of Pt‐S─POP; k) *x*–*y* line scan profile, measured from Figure S7.

The local structure and coordination environment of M2 and Pt‐S─POP were examined using X‐ray absorption near‐edge structure (XANES) and Fourier‐transformed extended X‐ray absorption fine structure (FT‐EXAFS) spectroscopy. Pt L₃‐edge XANES analysis, compared with Pt foil (Figure 1f), indicates that the charge state of Pt in M2 and Pt‐S─POP is close to Pt(0). FT‐EXAFS spectra (Figure 1g) show a prominent peak at ≈1.95 Å in R space for both M2 and Pt‐S─POP, corresponding to Pt‐S and Pt‐P bonds. A peak at ≈2.63 Å, characteristic of Pt‐Pt bonding, is observed, confirming the atomic dispersion of Pt. Further coordination analysis of Pt‐S─POP through fit analysis (Figure 1h,i) reveals a primary peak at 1.92 Å and a secondary peak at 1.86 Å, corresponding to Pt–S and Pt–P coordination, respectively. The derived structural parameters suggest a possible Pt‐S─POP coordination configuration (Table S2, Supporting Information). To further illustrate the atomic coordination of Pt, wavelet transform (WT) analysis was performed, offering distinct resolution in both R and k spaces. WT contour plots of Pt‐S─POP display a significant intensity at ≈6.7 Å, similar to M2, resulting from Pt‐light atom scattering. In contrast, WT plots of Pt foil exhibit a peak intensity at 10.2 Å, attributed to Pt–Pt contributions, reinforcing the atomic dispersion of Pt single atoms (**Figure** **2**j). TGA (Figure S9, Supporting Information) confirms that the POP framework remains stable up to 532.6 °C, with a 5% weight loss (*T_d5_*). After sulfurization, the *T_d₅_* of S─POP decreases to 390.6 °C. Pt‐S─POP exhibits a higher *T_d₅_* of 433.8 °C and produces more char, reflecting the enhanced stability imparted by SAC and co‐ligand coordination.

Density functional theory (DFT) calculations were conducted to investigate the reaction potential of thianthrene with sulfur species and to elucidate the coordination behavior of tetrathiocine ligands with a single Pt atom. As illustrated in Figure S3 (Supporting Information), the initial thianthrene precursor undergoes a favorable transformation to generate an intermediate structure (M1) with a calculated total energy of −5.90 eV, indicating the thermodynamic stability of the core framework prior to metal coordination. To further explore the structural preference between different Pt coordination environments, we evaluated the reaction energies (∆*E*) associated with the formation of two representative single‐atom Pt sites: SS─Pt‐SS (type I) and SS─Pt‐PP (type II), corresponding to sulfur‐only and sulfur‐phosphorus co‐coordinated configurations, respectively. As shown in Figure S3 (Supporting Information), the transformation from thianthrene to the type II structure exhibits a reaction energy of −1.10 eV, which is notably lower than that for the formation of type I (−0.75 eV). These results suggest that the SS─Pt‐PP (type II) coordination is thermodynamically more favorable, likely due to the additional electronic stabilization provided by the phosphorus ligands. Consequently, type II may represent the predominant coordination motif in the final Pt‐S─POP structure synthesized under experimental conditions. This thermodynamic tendency aligns with the experimental observation of successful P‐containing coordination environments in the final catalyst material.

The X‐ray diffractometer (XRD) analysis (Figure 2a) shows that the thianthrene‐based POP is amorphous. This characteristic remains largely unchanged after sulfurization and the removal of excess sulfur, indicating the absence of crystalline sulfur impurities in S─POP. Following Pt coordination, a broader reflection appears at 2*θ* = 4.9–9.3°, suggesting weakened interlayer interaction and potential layer extension due to Pt and phosphine co‐ligand coordination. The synthesized POP exhibits nanoporosity, with pore widths of 0.4–2.9 nm, as determined by the Barrett–Joyner–Halenda (BJH) method. Additionally, it has a Brunauer–Emmett–Teller (BET) surface area of 194.4 m^2^ g⁻¹, calculated via N₂ physisorption (FigureS4, Supporting Information). After sulfurization, C─S─C bonds transform into C─S─S─C bonds, expanding pore widths to 3.5 nm and increasing the BET surface area to 301.5 m^2^ g⁻¹. Replacing some C─S─S─C linkages with Pt(PPh₃)₄ slightly reduces pore widths (0.3–3.2 nm) and BET surface area (282.4 m^2^ g⁻¹) (Figure 2b,c). As shown in Figure S5 (Supporting Information), synthesized POPs and their derivatives exhibit varying degrees of agglomeration or stacking, resulting in irregular shapes and patterns in scanning electron microscope (SEM) images. SEM‐energy‐dispersive X‐ray (EDX) mapping of Pt‐S─POP shows uniform sulfur and Pt distribution, confirming high Pt loading in the carbon framework. Furthermore, transmission electron microscope (TEM) images reveal a distinct elliptical and sheet‐like structure (Figure 2d,e, Figure S6, Supporting Information). To clarify the SAC structure, we conducted high‐angle annular dark‐field (HAADF) scanning transmission electron microscopy (STEM) on Pt‐S─POP to visualize individual Pt metal atoms. Figure 2f shows well‐dispersed bright spots corresponding to SAC, confirmed by energy‐dispersive X‐ray spectroscopy (EDS) analysis in STEM mode (Figure 2g–j). Additionally, EDS analysis shows that yellow contrast spots are surrounded by S and P species, confirming Pt coordination with benzenediothiolate and phosphine ligands (Figure S7, Supporting Information). Figure S8 (Supporting Information) shows the EDS elemental analysis results, confirming the presence of P, S, and Pt in the structure, which is consistent with the proposed coordination environment in Pt‐S─POP. TGA was performed in the 50–800 °C range under N₂ to evaluate the thermal stability of POPs (Figure S9, Supporting Information). The POP framework remained stable up to 532.6 °C, where a 5% weight loss was observed, indicating its stability under sulfurization conditions. After sulfurization, the *T_d₅_* of S─POP decreased to 390.6 °C due to the formation of C─S─S─C bonds. Notably, Pt‐S─POP exhibited a higher *T_d₅_* of 433.8 °C and produced more char, attributed to the partial replacement with coordinated SAC and co‐ligand.

### 2.2 Activity and Catalytic Conversion on Pt‐S─POP

**Figure 3:**
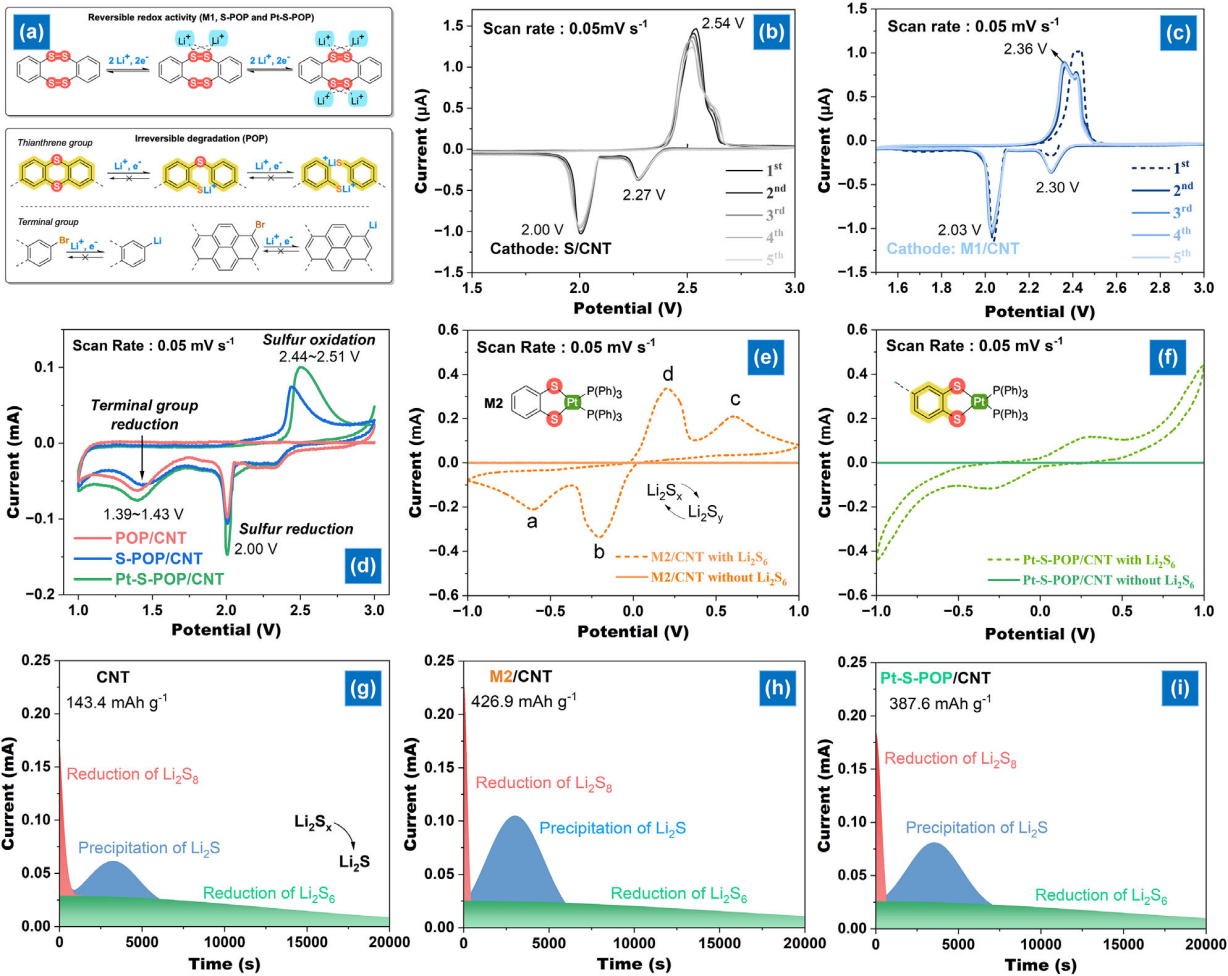
a) Plausible degradation pathway and reversible Li–ion interaction in POP‐based materials; CV curves of cells with b) S/CNT, c) M1/CNT, d) POP/CNT, S─POP/CNT, and Pt‐S─POP/CNT cathodes; CV curves of symmetric cells with e) M2/CNT and f) Pt‐S─POP; Potentiostatic Li_2_S nucleation profiles at 2.05 V on g) CNT, h) M2/CNT, and i) Pt‐S─POP/CNT electrodes.

Previous research has shown that the presence of a tetrathioncine linker in COFs induces cathodic activity against Li metal within the higher potential range of 1.2–2.8 V.^[^ ^14^ ^]^ However, COFs contain both C─S─S─C linkers and grafted polysulfide chains, exhibiting distinct and reversible redox peaks in CV measurements using a Li metal reference electrode. To better understand the redox behavior of the C─S─S─C linker specifically in S─POP and Pt‐S─POP, the M1 model compound was investigated (**Figure** **3**a). CV analysis of a sulfur/CNT composite with Li metal shows a strong oxidation peak at 2.54 V and reduction peaks at 2.27 and 2.00 V (Figure 3b), characteristic of Li‐S battery redox reactions. When M1 is combined with 10 wt.% CNT, the M1/CNT cathode exhibits similar redox activity with a reduced potential gap of 0.06 V (Figure 3c), indicating that the C─S─S─C structure enables reversible Li–ion interaction via the lone pair electrons in the S─S linkage (Figure 3a). For the POP/CNT hybrid cathode, irreversible reduction peaks at 1.40, 2.00, and ≈2.30 V appear in the first cycle but disappear in subsequent cycles (Figure S10a, Supporting Information). These degradation events, occurring at 1.40 V and higher voltages (2.00 V and ≈2.30 V), are likely due to C─Br and C─S bond cleavage, along with carbonate electrolyte decomposition caused by bromophenyl and LPS species (Figure 3a). Similarly, the first cycle of both S─POP and Pt‐S─POP exhibits irreversible degradation of terminal groups, which is absent in the second cycle (Figure S10b,c, Supporting Information). The C─S─S─C structure in both POPs demonstrates reversible Li–ion interaction via the lone pair electrons in S─S bonds, mirroring the redox process in M1 (Figure 3d). However, the porous structure weakens the affinity between C─S─S─C and Li‐ions, slowing the redox process. These results suggest that Pt‐S─POP readily interacts with Li‐ions, making it an effective separator modifier. Despite the presence of chemically bound sulfur in the S─POP and Pt‐S─POP framework, its impact on the overall capacity is minimal, attributed to the exceedingly low coating mass (≈0.04 mg cm⁻^2^) and sulfur content (≈0.004 mg cm⁻^2^), representing merely ≈0.1–0.2% of the cathode sulfur loading (2–4 mg cm⁻^2^). Consequently, its capacity contribution was not deducted in the performance assessment.

Beyond the electrochemical reaction of C─S─S─C in POPs, the electrocatalytic activity of LPS conversion was evaluated via CV using symmetrical cells with identical working and counter electrodes.^[^ ^12^ ^]^ Upon the addition of Li₂S₆ to the electrolyte, the CV curve of the cell with M2 (Figure 3e) displays four distinct redox peaks (a, b, c, d), corresponding to the reduction of Li₂S₆ → Li₂S₂/Li₂S, elemental S → Li₂S₆, and the oxidation of Li₂S₂/Li₂S → Li₂S₆ and Li₂S₆ → elemental S, respectively.^[^ ^15^ ^]^ These results indicate that the M2 SAC exhibits significant catalytic activity. However, the use of Pt‐S─POP leads to a slight reduction in the reversible transformation of LPSs, primarily due to the lower SAC loading within the porous structure (Figure 3f and Figure S11, Supporting Information). Nevertheless, the Pt‐based SAC effectively catalyzes LPS conversion, as confirmed by analyses of Li₂S₂/Li₂S nucleation and dissolution.^[^ ^16^ ^]^ Figure 3g–i presents the discharge profiles of the potentiostatic process associated with Li₂S₂/Li₂S formation for CNT, M2/CNT, and Pt‐S─POP/CNT electrodes. The redox peaks in Pt SAC‐based cells appear significantly earlier than in CNT‐based cells, and the nucleation capacities of M2/CNT (426.9 mAh g⁻¹) and Pt‐S─POP/CNT (387.6 mAh g⁻¹) are notably higher than CNT (143.4 mAh g⁻¹). These results confirm the superior out‐of‐plane catalytic activity of Pt‐based SAC in facilitating redox reactions between soluble Li₂S_n_ and solid Li₂S₂/Li₂S. Although M2 exhibits higher intrinsic catalytic activity, its small‐molecule nature results in partial dissolution in the electrolyte during cycling, which compromises long‐term stability. This inherent drawback limits its feasibility as a functional separator coating. In contrast, Pt‐S─POP offers a structurally robust platform that effectively immobilizes Pt active sites, ensuring both catalytic activity and practical applicability in working cells.

### 2.3 Electrochemical Performance of Li‐S Batteries Constructed using Pt‐S─POP

The electrochemical performance of Li‐S batteries using Pt‐S─POP was optimized by incorporating large‐size graphene oxide (LGO) to enhance the structural control of ultra‐thin coatings.^[^ ^16^ ^]^ LGO, prepared via pH‐assisted selective sedimentation, was combined with synthesized POPs through its pyrene skeleton to form a uniform coating layer.^[^ ^17^ ^]^ The GO‐based slurries were applied to a PE separator and reduced with HI‐AcOH, resulting in a smooth, strongly adhesive coating (Figure S12, Supporting Information). Spectral analysis (Figures S13 and S14, Supporting Information) confirmed reduced oxygen content. Cross‐sectional SEM images (Figure S15a,b, Supporting Information) showed ion‐sieving layers of ≈120 nm (rGO) and ≈160 nm (Pt‐S─POP/rGO) with an ultra‐light weight of ≈0.04 mg cm⁻^2^. The PE surface's irregular pores (50–130 nm, Figure S16, Supporting Information) were fully covered, forming a uniform, compact coating (Figure S15c,d, Supporting Information). Additionally, the rGO‐coated layer enhanced thermal stability at 150 °C (Figure S17, Supporting Information).

This study analyzed the electrolyte absorption capacity and wettability by measuring electrolyte uptake (*EU*), electrolyte retention (*ER*), and contact angle. As shown in Figure S18 (Supporting Information), modifying the pristine PE separator with rGO increased its *EU* from 78.6% to 147.1%, attributed to rGO's improved electrolyte affinity. The porous nature of POPs further enhanced absorption capacity, raising *EU* beyond 188%. This effect was also reflected in the increased *ER* across different deposition periods. Additionally, electrolyte droplets spread more on the modified separators (contact angles <50°), whereas the pristine PE exhibited a larger contact angle of 89.2° (Figure S19, Supporting Information). These results indicate that incorporating a porous structure enhances separator‐electrode compatibility and facilitates Li‐ion diffusion. For evaluating the permeation resistance of POP‐based modifiers against soluble LPSs, a two‐bottle test was conducted with PE and Pt‐S─POP/rGO‐modified separators sandwiched between them. Over time, Li₂S₆ diffusion into the pure electrolyte was clearly observed with PE but was nearly negligible with the Pt‐S─POP/rGO modifier. Even after 24 h (Figure S20, Supporting Information), Li₂S₆ migration remained insignificant, demonstrating the superior LPS─blocking ability of the POP/rGO hybrid layer. The electrochemical stability window of different separators was evaluated using linear sweep voltammetry (LSV) from 0 to 6 V at a scan rate of 10.0 mV s⁻¹. As shown in Figure S21 (Supporting Information), the oxidation current for PE peaks at 4.2 V, while modification with rGO and its hybrids enhances electrochemical stability. Figure S22 (Supporting Information) presents the electrochemical impedance spectroscopy (EIS) plots of SS|separator|SS symmetrical cells. The pure PE separator exhibits an ionic conductivity (*σ*) of 0.35 mS cm⁻¹, which increases to >0.51 mS cm⁻¹ with POP due to its improved electrolyte absorption and wettability.

**Figure 4:**
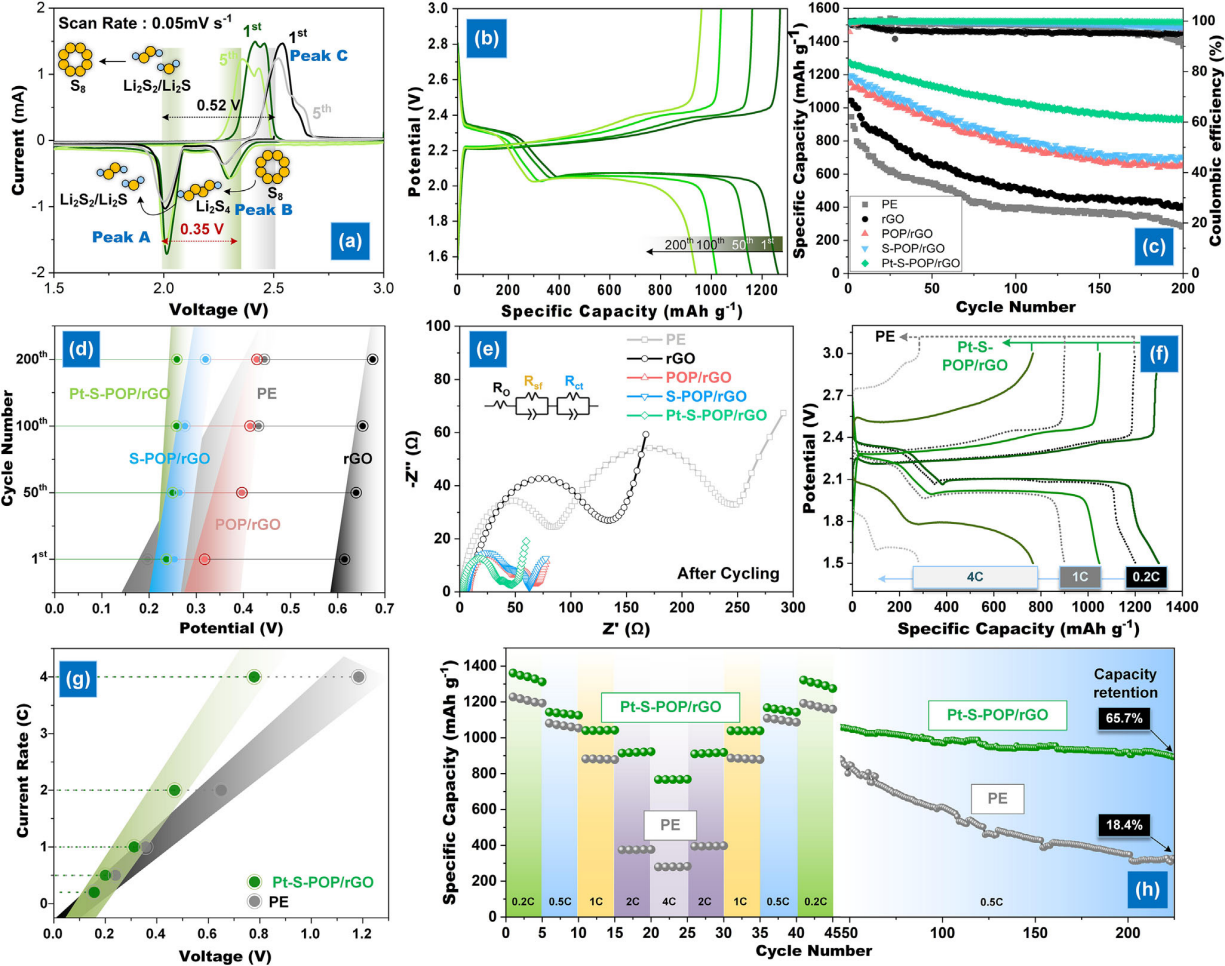
a) CV curves of the cell with a PE separator over 5 consecutive cycles; b) Galvanostatic discharge profiles of cell with Pt‐S─POP/rGO at 0.5 C; c) Cycling performance of cells with various separators at 0.5 C; d) Potential gap of cell at 50% discharge capacity with various separators at 0.5 C; e) EIS of cells with various separators at 0.5 C; f) Charge–discharge profiles, g) potential gap at 50% discharge capacity, and h) rate performance of cells assembled with PE and Pt‐S─POP/rGO separators under various current densities.

The CV profile reveals the typical multi‐step reaction mechanism in Li‐S batteries (**Figure** **4**a). The Pt‐S─POP/rGO‐modified separator exhibited the smallest redox peak potential difference and significantly lower Tafel slopes in the second reduction (Peak A, 34.6 mV dec⁻¹) and initial oxidation (Peak C, 50.2 mV dec⁻¹) (Figure S23, Supporting Information), indicating enhanced LPS redox kinetics. Figure S24 (Supporting Information) and Figure 4b present the galvanostatic discharge‐charge profiles, showing that the Pt‐S─POP/rGO‐modified separator achieves the lowest polarization potential and a high capacity of 1266.8 mAh g⁻¹ at 0.5 C. This performance surpasses PE, POP/rGO, and S─POP/rGO separators due to improved sulfur utilization. After 200 cycles (Figure 4c), the Pt‐S─POP/rGO separator retains 73.6% of its capacity, significantly higher than PE (30.6%), rGO (38.4%), POP/rGO (56.5%), and S─POP/rGO (59.2%). This is further confirmed by changes in the upper and lower discharge plateaus (*Q*_H_ and *Q*_L_, Figure S25, Supporting Information). Additionally, the Pt‐S─POP/rGO‐modified cell exhibits a reduced potential gap of <0.25 V at 50% discharge capacity (Figure 4d), nearly half that of the PE‐based cell. In contrast, a separator modified with rGO shows a larger potential gap, highlighting the benefits of the porous POP‐based modifier for redox reactions during cycling. These findings align with the CV profiles and low interface resistances in the EIS spectra (Figure S26, Supporting Information and Figure 4e). The EIS results also reveal that the Pt‐S─POP/rGO‐modified cell exhibits smaller semicircles at low and medium frequencies, corresponding to lower charge transfer resistance (*R*_ct_) and improved Li⁺ migration through the passivation film.^[^ ^7^, ^18^ ^]^ This suggests superior cross‐interface transport and thinner passivation layers on both the cathode and anode surfaces.

### 2.4 Impacts of Pt‐S─POP in Battery Performances

To comprehensively assess the impact of coordination control of SACs on the rate and long‐term cycling performance of Li‐S batteries, rate performance was evaluated at 0.2, 0.5, 1, 2, and 4 C (Figure 4f–h). The Pt‐S─POP/rGO‐modified separator achieved high discharge capacities of 1360, 1146, 915, 764, and 586 mAh g⁻¹, which significantly exceeded those of the PE separator (1229, 1081, 884, 380, and 279 mAh g⁻¹, respectively). In addition, the corresponding charge–discharge voltage profiles at various current densities (Figure 4f) reveal distinct differences in polarization behavior. The Pt‐S─POP/rGO cell maintains more defined and stable voltage plateaus (Figure 4g), with smaller potential gaps between charge and discharge compared to the PE‐based cell, indicating lower polarization and improved electrochemical kinetics. As shown in Figure 4f, the Pt‐S─POP/rGO separator preserves two clear reduction plateaus and one oxidation plateau across all tested current densities, whereas the PE cell exhibits increasing overlap and fading of these features at higher rates‐signs of sluggish conversion dynamics. Moreover, the improved voltage stability at high current densities reflects more efficient liquid–solid phase conversion (Li₂S₆ → Li₂S₂/Li₂S and vice versa), further supported by the nucleation‐related capacity retention. After rate‐varying cycling, the subsequent recovery cycling at 0.5 C shows that the Pt‐S─POP/rGO cell retained 65.7% of its original capacity, while the PE cell retained only 18.4% (Figure 4h), highlighting superior reversibility and structural integrity associated with the catalytic function of Pt‐SACs.

**Figure 5:**
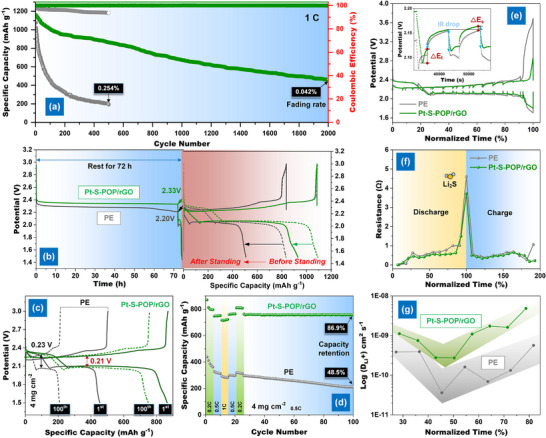
a) Long‐term cycling performance at 1 C and b) 26th cycled voltage‐time profiles at 0.5 C for cells with PE and Pt‐S─POP/rGO separators; c) cycle performance and d) rate performance of high S─loading cells with PE and Pt‐S─POP/rGO separators at 0.5 C; e) GITT profiles, f) IR drop, and g) calculated Li ion diffusion coefficient of the cells with PE and Pt‐S─POP/rGO separators.

As shown in **Figure** **5**a, the Pt‐S─POP/rGO‐modified separator significantly improves cycling stability, maintaining a low‐capacity fading rate of 0.030% per cycle over 2000 cycles at 1 C. However, under prolonged cycling at 0.5 C, the cell with a PE separator exhibits a higher degradation rate of 0.254% per cycle after 300 cycles, primarily due to the severe LPS shuttle and sluggish redox kinetics. To further investigate the suppression of the shuttle effect, we examined the morphology of the Li metal anode and the appearance of the separators after cycling. As shown in Figure S27 (Supporting Information), the Li anode retrieved from the PE‐based cell displays obvious signs of surface corrosion and the presence of irregular particle deposits, indicating severe polysulfide‐induced side reactions. In contrast, the Li anode from the Pt‐S─POP/rGO‐modified cell remains smooth and uniform, and the separator surface is intact and clean, confirming the ability of the functional coating to inhibit polysulfide migration and preserve interfacial stability. Overall, the enhanced long‐term performance and structural preservation of the Pt‐S─POP/rGO‐modified cell outperform previously reported ion‐sieving membranes based on GO or rGO (Figure S28, Table S3, Supporting Information), further validating the effectiveness of our design.

To evaluate the practical applications of Li‐S batteries, we examined the effects of self‐discharge and high sulfur loading, both strongly influenced by the shuttle effect. Figure 5b presents the open‐circuit voltage (OCV) measured continuously over 72 h. During this period, the Pt‐S─POP/rGO cell exhibited greater OCV stability, with a final voltage of 2.33 V compared to 2.20 V for the PE cell, indicating superior resistance to self‐discharge. Additionally, the Pt‐S─POP/rGO cell showed significantly lower capacity loss (161.4 mAh g⁻¹) than the PE cell (327.1 mAh g⁻¹). In high‐S loading tests, it achieved an initial area capacity of 1.8 mAh cm⁻^2^ and retained 86% of its capacity after 100 cycles, outperforming the PE cell, which started at 0.9 mAh cm⁻^2^ and retained only 45%. These results confirm that the Pt‐S─POP/rGO‐modified separator effectively mitigates the shuttle effect and enhances LPS redox kinetics, even under high sulfur loading conditions. This advantage is also evident in the rate performance of high‐S loading cells, which show significantly higher capacity and retention than PE (Figure 5d).

To illustrate the efficiency of the Pt‐S─POP/rGO‐modified separator under lean electrolyte conditions, rate performance evaluations were performed at an E/S ratio of 5 µL mg⁻¹ throughout a range of 0.2–2.0 C (Figure S29, Supporting Information). Notably, the Pt‐S─POP/rGO cell retained a high capacity of 525 mAh g⁻¹ at 2.0 C, compared to only 108 mAh g⁻¹ for the PE‐based cell. The Pt‐S─POP/rGO consistently demonstrated superior performance across all rates, highlighting its capacity to maintain redox kinetics and sulfur usage with less electrolyte. The charge–discharge profiles under these conditions exhibited more stable and pronounced voltage plateaus for the Pt‐S─POP/rGO cell. Additionally, the voltage gap at 50% discharge was smaller than that of the PE cell, indicating reduced polarization and improved energy efficiency. This enhanced performance is attributed to the Pt‐S─POP's porous structure and the catalytic role of atomically dispersed Pt sites. Together, these features improve electrolyte absorption and promote rapid LPS conversion, enabling efficient operation even under electrolyte‐constrained conditions.

Efficient Li⁺ diffusion during cycling is a crucial prerequisite for the effective conversion of LPSs. The galvanostatic intermittent titration technique (GITT) was used to analyze the redox reaction and diffusion kinetics in cells with PE and Pt‐S─POP/rGO‐modified separators. Figure 5e presents the GITT profiles, where the potential hysteresis during the relaxation period corresponds to the excess potential of the polylithiation platform (Supporting Information). The results show that Pt‐S─POP/rGO‐modified separators exhibit a lower Δ*V* than PE‐based cells, indicating reduced overpotential during repeated charge–discharge cycles. Additionally, the IR drop variation (Figure 5f) further confirms the significantly reduced polarization and enhanced electrochemical kinetics in the Pt‐S─POP/rGO cell. At different voltage levels, this material substantially improves Li⁺ diffusion compared to PE (Figure 5g). The enhanced Li⁺ diffusion can be attributed to two key factors: i) the stable POP framework with an optimal pore size, which creates numerous ion diffusion channels and promotes efficient electrolyte flow, and ii) the abundant coordinated Pt atoms, which lower the energy barrier for LPS conversion, reducing Li⁺ diffusion resistance and facilitating migration.

**Figure 6:**
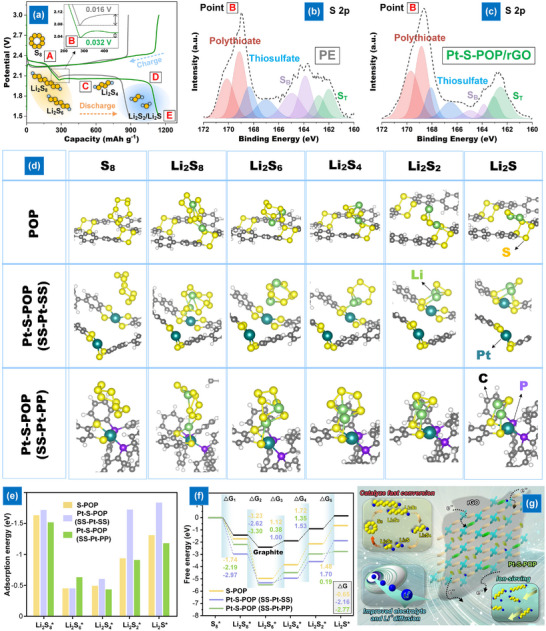
a) Charge‐discharge profile of cells with PE and Pt‐S─POP/rGO separators; b,c) XPS analysis of discharge products at point B; d) Simulated charge density of S─POP and Pt‐S─POP; e) Binding energy of S species on S─POP and Pt‐S─POP; f) Energy profiles for LPS reduction and Li₂S decomposition; g) Schematic of the Pt‐S─POP‐based ion‐sieving layer mechanism.

Extensive research is being conducted on the electrocatalytic performance and mechanism of LPS electrocatalysis on Pt‐S─POP/rGO separator using XPS analysis from the disassembled cells at point B in **Figure** **6**a. The charge‐discharge profile revealed that the cell containing Pt‐S─POP/rGO has a significantly lower potential barrier at Point B, suggesting that the activation energy for the conversion of Li_2_S_n_→Li_2_S_2_/Li_2_S is greatly reduced in this cell. In the XPS S2p analysis of Figure 6b reveals two distinct peaks at 169.7/168.8 and 168.1/166.6 eV are assigned to R‐SO_2_‐R/SO_4_^2^^−^ and S_2_O_3_ ^2^ ^−^/SO_3_^2^^−^, respectively, resulting from the oxidization of sulfur species either during the transfer of sample or due to the residual presence of LiTFSI. The Pt‐S─POP/rGO modification significantly increases the area ratio (*S*_T_^−1^/*S*_B_^0^) from 0.65 to 3.13, corresponding to the bridging sulfur (*S*_B_^0^) and terminal sulfur (*S*_T_^−1^) at 164.9/163.9 and 163.0/162.6 eV, respectively. This suggests that the presence of SAC during the liquid–liquid transition process leads to an increased production of short‐chain LPSs. Thus, the Pt‐based SAC within POP exhibits exceptional catalytic activity toward dissolved LPSs, facilitating their conversion into solid LPSs without the need for shuttling through the separator. The lower dissociation energy of Li_2_S_4_ and Li_2_S_2_ on the surface of Pt‐S─POP leads to enhanced nucleation and growth of Li_2_S, resulting in improved redox kinetics and increased sulfur utilization during cycling.

First‐principles computational simulations using DFT were performed to gain a deeper understanding of the adsorption and reaction mechanisms of LPSs at the molecular or atomic level. Figure S30 (Supporting Information) presents the optimized adsorption configurations for different LPSs on the S─POP and Pt‐S─POP substrate with two different atomic Pt sites (denoted as SS─Pt‐SS and SS─Pt‐PP moieties) (Figure S31, Supporting Information). Figure 6e demonstrates that the regulating effect of S coordination on the d orbital electrons of Pt single atoms amplifies d–p orbital hybridization with sulfur atoms in LPSs, resulting in elevated adsorption energies of SS─Pt‐SS moieties for LPSs, beyond those of S─POP. Despite the SS─Pt‐PP moieties facilitating P and S co‐coordination on the d orbital electrons of Pt single atoms, it exhibits reduced adsorption energy for LPSs due to an increased spatial barrier created by the surrounding phenyl group of PPh_3_. The improved adsorption characteristics of Pt‐S─POP correspond with the observed adsorption test (Figure S20, Supporting Information).

First‐principles computational simulations using DFT were performed to gain a deeper understanding of the adsorption and reaction mechanisms of LPSs at the molecular or atomic level. Figure S30 (Supporting Information) presents the optimized adsorption configurations for different LPS species on the S─POP and Pt‐S─POP substrates, which feature two types of atomic Pt coordination environments: SS─Pt‐SS (type I) and SS─Pt‐PP (type II) moieties, as illustrated in Figure S31 (Supporting Information). As shown in Figure 6e, the S─coordination environment in SS─Pt‐SS modulates the d orbital electrons of the Pt single atoms, enhancing d–p orbital hybridization with the sulfur atoms in LPSs. This interaction results in higher adsorption energies compared to S─POP, indicating a stronger binding affinity for LPS intermediates. Although the SS─Pt‐PP moiety enables both P and S co‐coordination around Pt, it shows relatively lower adsorption energy due to the steric hindrance caused by the bulky phenyl groups from PPh₃, which creates a spatial barrier that limits effective orbital overlap. These theoretical findings correlate well with the experimental results observed in the LPS adsorption test (Figure S20, Supporting Information) and together provide insights into how the coordination environment of single‐atom Pt species governs both the adsorption behavior and the structural evolution within the porous polymer network.

Furthermore, Gibbs free energy (Δ*G*) was calculated for the S reduction process to evaluate the thermodynamic propensity of LPSs. In Figure 6f, the Gibbs free energy of S─POP is considerably lower than that of pure graphite across all reaction stages, suggesting that the tetrathiocine linker in POPs enhances the reaction kinetics of LPSs. Through the coordination of Pt, it is evident that the Gibbs free energy of S─POP (−0.65 eV) was markedly decreased to less than −2.16 eV for Pt‐S─POP. The reduction of Li_2_S_2_ to Li_2_S is acknowledged as the rate‐limiting stage in the discharge process.^[^ ^19^ ^]^ The reduced Gibbs free energy (0.19 eV) in type II moieties for this phase, in contrast to the type I model (1.70 eV), indicates that the formation of Li₂S is thermodynamically advantageous on Pt SAC with an out‐of‐plane architecture. The incorporation of a tetrathiocine linker and the synergistic effects of both moieties, Pt SAC in porous polymer, facilitate rapid ion transfer, strong interactions, and catalyst effects with LPSs, hence expediting liquid‐solid deposition on the Pt‐S─POP/rGO modified layer. Based on comprehensive experimental analyses and theoretical calculations, the multifunctional mechanism of the Pt‐S─POP/rGO layer was postulated and depicted in Figure 6g. The tetrathiocine‐linked POP exhibits improved affinity and stable solvation characteristics, along with physical and chemical barriers for soluble LPSs, facilitating quick Li‐ion conduction and significant retardation of LPSs. The introduction of Pt SAC into the separator markedly enhances the nucleation and development of Li_2_S_2_/Li_2_S, resulting in more effective redox kinetics and increased sulfur utilization over prolonged charge–discharge cycles.

## 3 Conclusion

This research emphasizes a notable improvement in Li‐S battery architecture with the novel incorporation of out‐of‐plane SACs within POPs. Utilizing a tetrathiocine coordination motif, we attained a distinctive spatial configuration that both stabilizes the active materials and improves the catalytic efficiency for LPS conversion. The Pt‐S─POP/rGO separator demonstrates remarkable capabilities in mitigating the LPS shuttling effect and accelerating redox kinetics, crucial for optimizing battery performance. This unique out‐of‐plane coordination facilitates efficient interaction between the metal centers and LPSs, leading to enhanced kinetics in liquid‐solid transformations. As a result, the separator exhibits excellent cycling stability, capacity retention, and self‐discharge resistance, even under high sulfur loadings. These findings highlight the significance of unique chemical structures in improving the electrochemical performance of Li‐S batteries, facilitating the development of advanced energy storage systems with increased energy densities and extended cycling lifetimes. This material design approach, leveraging the unique properties of SACs, significantly contributes to advancing the field of Li‐S battery technology.

## 4 Experimental Section

### Material Synthesis

i) Synthesis of Py‐4Br: Pyrene (1.0 g) was dissolved in nitrobenzene, and bromine (1.15 mL) was added dropwise. The mixture was heated at 120 °C for 4 h, then cooled, filtered, and washed with ethanol, yielding Py‐4Br (91%) as a green solid. ii) Synthesis of Th‐2Br: Thianthrene (2.16 g) was dissolved in acetic acid, followed by bromine (4 mL) addition. The mixture was heated at 80 °C for 16 h, then cooled and precipitated with deionized water. The product was washed, recrystallized, and obtained as Th‐2Br (88%). iii) Synthesis of S─Th (M1): Thianthrene (2.0 g) was mixed with sulfur (10.0 g) in a flame‐sealed quartz ampoule and heated at 180 °C for 6 h. The product was washed with toluene, recrystallized, and dried at 70 °C, yielding M1 (13%). iv) Synthesis of Pt‐S─Th (M2): M1 (0.11 g) and Pt(PPh₃)₄ (0.98 g) were dissolved in xylene, stirred at 155 °C for 48 h, then washed and recrystallized in DCM. The brown powder M2 (40%) was obtained after drying at 70 °C. The details of the synthesis of 1,3,6,8‐Tetrabromopyrene (Py‐4Br) (Scheme S1, Supporting Information), 2,7‐Dibromothianthrene (Th‐2Br) (Scheme S2, Supporting Information), S─Th (M1) and Pt‐S─Th (M2) (Scheme S3, Supporting Information) are provided in the Supporting Information.

### Synthesis of POP, S─POP, and Pt‐S─POP

Py‐4Br, benzene‐1,4‐diboronic acid, Th‐2Br, Pd(PPh₃)₄, and K₂CO₃ were mixed in DMF/water, subjected to freeze‐pump‐thaw cycles, and heated at 120 °C for 72 h. The product was purified via Soxhlet extraction with THF and dried at 70 °C, yielding POP (64%). POP (0.8 g) was then mixed with sulfur (4.0 g) in a flame‐sealed quartz ampoule under vacuum and heated stepwise at 120 °C (2 h), 150 °C (6 h), and 360 °C (24 h). The product was washed with toluene and dried, yielding S─POP (20%). Finally, S─POP (0.15 g) was combined with Pt(PPh₃)₄ (0.2 g) in xylene, stirred at 155 °C for 48 h, followed by solvent exchange and drying to afford Pt‐S─POP. The details of the synthesis of POP, S─POP, and Pt‐S─POP are provided in the Supporting Information (Scheme S4, Supporting Information).

### Modification of the PE Separators

The preparation required combining a solution of 60 wt.% POP and 40 wt.% LGO in DMF, followed by ultrasonic agitation for 1 h and overnight stirring with an ARE‐310 planetary centrifugal mixer to yield a slurry. The slurry was applied to commercial PE separators utilizing a doctor‐blade coating technique with a thickness of 10 µm, and then dried at 60 °C to produce a POP/GO modified separator. Thereafter, reduction is carried out utilizing hydrogen iodide (HI) to transform GO into rGO. Subsequent to this reduction phase, extensive washing with deionized water and ethanol is performed, followed by drying at 60 °C to produce POP/rGO. GO, S─POP/GO, and Pt‐S─POP/GO were synthesized using identical procedures and subsequently reduced to rGO, S─POP/rGO, and Pt‐S─POP/rGO, respectively.

### Separator Properties

The specifics of the measurement for thermal shrinkage ratio, electrolyte uptake, electrolyte retention, and ionic conductivity are included in the Supporting Information.

### Assembly of Symmetric Dummy Cells

Li₂S and sulfur, in amounts corresponding to the nominal stoichiometry of Li₂S₆, were added to a 1:1 (v/v) DOL/DME mixture and stirred overnight at 50 °C, yielding a 0.2 mol L⁻¹ Li₂S₆ solution. CNT‐based electrodes were punched into 10.0 mm diameter disks, and 0.35 mg cm⁻^2^ of M2 and Pt‐S─POP powders were loaded onto these disks, forming M2‐based and Pt‐S─POP‐based electrodes, respectively. The electrode preparation involved ultrasonically dispersing M2 and Pt‐S─POP powders in NMP, dropwise coating the CNT‐based electrodes with the suspension, and drying at 55 °C for 12 h. Two identical electrodes were assembled into a standard 2032 coin cell, with 20.0 µL of Li₂S₆ electrolyte added.

### Measurement of Li_2_S Nucleation

A 0.2 mol L⁻¹ Li₂S₈ solution was prepared as the electrolyte by dissolving stoichiometric amounts of lithium sulfide and sulfur powder in tetraglyme under vigorous magnetic stirring. CNT‐based electrodes were punched into 10 mm diameter disks and used as current collectors, with 0.5 mg of M2 and Pt‐S─POP powders loaded for cell assembly. Li foil served as the anode. For cell preparation, 20 µL of Li₂S₈ electrolyte was first added to the cathode, followed by 20 µL of electrolyte without Li₂S₈ in the Li anode compartment. The assembled cells were galvanostatically discharged at 0.112 mA to 2.06 V, then potentiostatically discharged at 2.05 V to promote Li₂S nucleation and growth. The potentiostatic discharge was terminated when the current dropped below 10⁻⁵ A.

### Cell Assembly and Electrochemical Measurement

The battery assembly takes place inside a glove box filled with argon, with H_2_O < 0.5 ppm and O_2_ < 0.5 ppm. Before battery assembly, the separator is sliced into circular discs measuring 16 mm in diameter. CR2032 coin cells are used, with the components arranged in the following sequence: shell, S─cathode, electrolyte, separator, Li metal, spacer, spring, and shell. Finally, the assembled battery is sealed using a battery sealing machine. The electrolyte‐to‐separator (E/S) ratio is ≈20 µL mg^−1^.

### Computational Detail

The Vienna Ab initio Simulation Package (VASP) 5.4.4 code with the Perdew–Burke–Ernzerhof (PBE) functional performed all DFT calculations in the present work.^[^ ^20^ ^]^ The ion–electron interaction was described by the projector augmented wave (PAW) method^[^ ^21^ ^]^ with a cutoff energy of 500 eV. The S─POP and Pt‐S─POP model with a 7 × 7 supercell were constructed to calculate the adsorption energy of lithium polysulfides (Li_2_S_n_). A k‐point mesh with a size of 3 × 3 × 1 and the spin polarization was applied for the calculations. Furthermore, the DFT‐D3 correction method in Grimme's scheme^[^ ^22^ ^]^ was used to accurately describe the long‐range vdW interactions. All the calculations were carried out until the total energy and force were less than 10^−5^ eV per atom and 0.05 eV Å^−1^, respectively. Finally, the adsorption energies (*E*_ads_) were defined as follows:

(1) $$E_{\mathrm{ads}}=E_{\frac{\mathrm{ad}}{\mathrm{sub}}}-E_{\mathrm{sub}}-E_{\mathrm{ad}}$$

where *E*_ad/sub_, *E*_ad_, and *E*_sub_ are the total energies of the optimized adsorbate (Li_2_S_n_)/substrate, the adsorbate in the gas phase, and the clean substrate, respectively. The free energies were acquired by *G*  = *E*_total_  + *E*_ZPE_ − *TS*, where *E*_total_, *E*_ZPE_, and *TS* are the ground‐state energy, zero‐point energies, and entropy terms, respectively, with the latter two taking vibration frequencies from DFT calculations.

## Conflict of Interest

The authors declare no conflict of interest.

## Supporting information



Supporting Information

## Data Availability

The data that support the findings of this study are available in the supplementary material of this article.
