# Supplementary material for: Single‐Atom Catalyst‐Integrated Porous Organic Polymers for High‐Performance Lithium‐Sulfur Batteries
Source: Small. 2025 Jun 16;21(32):2503250. doi: 10.1002/smll.202503250 (PMC12366242; doi:10.1002/smll.202503250)
Supplement: Supplementary file 1 — Supporting Information [file SMLL-21-2503250-s001.docx]

Supporting Information

Single-Atom Catalyst-Integrated Porous Organic Polymers for High-Performance Lithium-Sulfur Batteries

Yun-Sheng Ye,* Mohamed Gamal Mohamed, Nai-Hua Ye, Ting-Yun Hung, Guan-Yu Chen, Shi-Hsin Lin, Meng-Che Tsai, Bing-Joe Hwang, and Shiao-Wei Kuo*

**S1. Experimental Section**

**S1.1. Materials**

Li sheets (battery level, 15 mm diameter, 1 mm thick), aluminum foil (≥ 99.7 %, 15 μm thick), polyvinylidene difluoride (PVDF, HSV900 type), were purchased from Shenzhen Kaishenzhi Technology Co., Ltd, bis(trifluoromethane)sulfonimide lithium salt (LiTFSI, >98%), sublimed sulfur (S) were purchased from Adamas Company, lithium hydrosulfide (Li_2_S, 99.5 %), graphite (powder, >99wt%, 0.5-3 μm) and lithium nitrate (LiNO_3_, 99 %) were purchased from LeYan Company, N-methyl-2-pyrroldone (NMP, ≥99%) and tetrahydrofuran (THF) were purchased from Aladdin Company, 1,3-dioxolane (DOL, 98 %) and 1,2-dimethoxyethane (DME, 99 %) were purchased from TCI Company, tetrakis(triphenylphosphine) palladium(0) [Pd(PPh_3_)_4_], dimethylformamide (DMF), thianthrene, nitrobenzene, and ethanol were purchased from Sigma-Aldrich Company. Polyethylene separator (PE, thickness 16 μm) was purchased from Alfa Chemistry Co., Ltd, pyrene, potassium carbonate (K_2_CO_3_), sulfuric acid (H_2_SO_4_), bromine (Br_2_), and benzene-1,4-diboronic acid were purchased from J. T. Baker, xylene, dichloromethane (DCM), methanol, and acetone were purchased from Alfa Aesar.

**S1.2. Materials characterization**

The Fourier transform infrared spectroscopy (FTIR) analysis was conducted using a Nicolet Avatar 320 FTIR spectrometer. X-ray photoelectron spectroscopy (XPS) analysis was performed utilizing a monochromatized Al Kα anode on the Axis Ultra DLD Kratos AXIS SUPRA instrument from Thermo Kalpha. Nuclear magnetic resonance (NMR) spectra were acquired using a Varian INOVA-400 MHz spectrometer operating at 400 MHz for ^1^H nuclei. Mass spectrometry analyses were conducted using the Impact HD Q-TOF mass spectrometer manufactured by Bruker in Germany. The ^13^C solid-state nuclear magnetic resonance (solid-state NMR) spectra were carried out using the Bruker Avance III HD 400MHz NMR Spectrometer. Elemental analyzer (EA) spectrometric analyses were conducted using the UNICUBE model from Elementar, Germany. Thermogravimetric analysis (TGA) was conducted using the Q50 instrument, with heating performed from 40 to 800 °C at a rate of 20 °C min^-1^ under a nitrogen (N_2_) atmosphere. The specific surface area was determined employing the Brunauer-Emmett-Teller (BET) method with the ASAP2420-4MP instrument from the USA. The scanning electron microscopy (SEM) and the energy-dispersive X-ray spectroscopy (EDX) elemental mappings of the samples were using the Gemini SEM 300 instrument from Germany. The contact angle (CA) was measured using the Dataphysics OCA 20 instrument, with the solution comprising an electrolyte. The transmission electron microscopy (TEM) analysis was carried out utilizing the Talos F200X G2 TEM. X-ray diffraction (XRD) patterns were acquired using a diffractometer equipped with Cu-Kα radiation (*λ* = 0.154 nm) on the Smart Lab-SE instrument from Japan. UV-visible spectra were acquired using a UV-visible spectrophotometer (Evolution 220, Thermo Scientific).

**S1.3. Synthesis and preparation**

***Synthesis of 1,3,6,8-Tetrabromopyrene (Scheme S1)*:** Pyrene (1.0 g) was mixed with nitrobenzene (10 mL) in a flask. Bromine (1.15 mL) was added dropwise to nitrobenzene (10 mL). The resulting mixture was subjected to heating at 120 °C for a duration of 4 h, subsequently cooled to 25 °C. Following filtration and ethanol washing, the product 1,3,6,8-Tetrabromopyrene (Py-4Br) was acquired as a green solid. The yield approximates 91%.


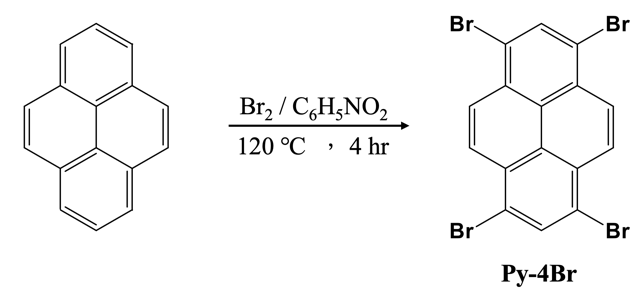


**Scheme S1.** Synthesis route of Py-4Br.

***Synthesis of 2,7-Dibromothianthrene (Scheme S2)*:** Thianthrene (2.16 g) was solubilized in acetic acid (40 mL) within a round-bottom flask, subsequently undergoing dropwise addition of bromine (4 mL). The mixture was subjected to heating at 80 °C and agitated for 16 h. Following cooling, 20 mL of deionized water was introduced, resulting in the formation of a white solid. The compound was washed with 5% NaHCO_3_ and recrystallized from MeOH and DCM, yielding 2,7-Dibromothianthrene (Th-2Br). The yield approximates 88%.


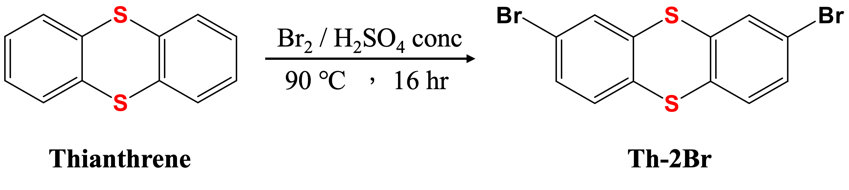


**Scheme S2.** Synthesis route of Th-2Br.

***Synthesis of S-Th (M1) (Scheme S3)*:** Thianthrene (2.0 g) was dispersed evenly in sulfur (10.0 g). The resultant mixture was placed into a quartz ampoule, which was then flame-sealed under vacuum conditions. The sealed ampoule was subjected to a regulated temperature protocol in a high-temperature reaction furnace, maintained at 180 °C for a duration of 6 h. After the reaction concluded, the resultant product was thoroughly washed through solvent exchange with toluene and subsequently recrystallized using MeOH. The resultant material was subsequently dried at 70 ^o^C, resulting in the achievement of the product, M1. The yield approximates 13%.

***Synthesis of Pt-S-Th (M2) (Scheme S3)*:** M1 (0.11 g) and Pt(PPh_3_)_4_ (0.98 g) were solubilized in xylene (20 ml). Xylene was carefully added dropwise to a round-bottomed flask, accompanied by continuous stirring at 155 °C for 48 h. A product of brown color was obtained. This product was rigorously washed through solvent exchange with xylene, subsequently undergoing recrystallization with DCM. The resultant material was subsequently dried at 70 ^o^C, resulting in the formation of a brown powder, M2. The yield is approximately 40%.

**Scheme S3**. Schematic illustration of the sulfurization and coordination of thianthrene resulting in the formation of M1 and M2 with covalently linked sulfur and coordinated Pt.

***Synthesis of POP (Scheme S4)*:** Py-4Br (1.0 g, 1.93 mmol), benzene-1,4-diboronic acid (0.64 g, 3.86 mmol), Th-2Br (0.22 g, 0.59 mmol), Pd(PPh_3_)_4_ (0.11 g), K_2_CO_3_ (5.52 g), DMF (20 ml), and deionized water (20 ml) were mixed together in a flame-dried Schlenk tube. The resultant mixture experienced three freeze/pump/thaw cycles. The mixture was then stirred and heated to 120 °C, sustaining this temperature for 72 h. Upon cooling to 25 °C, the mixture was subjected to rinsing with THF using a Soxhlet extraction apparatus. The resultant material was subsequently dried at 70 ^o^C, yielding a green-colored powder, POP. The yield approximates 64%.

**Scheme S4.** Synthesis of route for Pt-S-POP.

***Synthesis of S-POP (Scheme S4)*:** The synthesized POP (0.8 g) was dispersed within sulfur (4.0 g). The resulting mixture was placed inside a quartz ampoule, which was then flame-sealed under vacuum conditions. This sealed ampoule underwent a controlled temperature program within a tubular furnace, following this sequence: initially, the ampoule was held at 120 °C for 2 h, then at 150 °C for 6 h, and finally at 360 °C for 24 h. Upon completion of the reaction, the resulting product underwent thorough washing *via* solvent exchange with toluene to remove any unreacted sulfur. Finally, the resulting material was dried at elevated temperatures, ultimately resulting in the successful acquisition of the brown-colored powder, S-POP. The yield is about 20%.

***Synthesis of Pt-S-POP (Scheme S4):*** The synthesized S-POP (0.15 g) was incorporated into Pt(PPh_3_)_4_ (0.2 g). The resultant material was transferred into a round-bottom flask. Xylene was gradually introduced to the round-bottom flask while maintaining continuous stirring at 155 °C for a duration of 48 h. The resultant product was subjected to extensive washing *via* solvent exchange with xylene. Ultimately, the resultant material was subjected to drying at elevated temperatures, leading to the successful production of a brown powder, Pt-S-POP. The yield is approximately 45%.

***Preparation of Li_2_S_6_ and Li_2_S_8_ solution:*** Li_2_S and sulfur at a molar ratio of 1:5 and 1:7 was added to mixed DOL and DME in a 1:1 volume in a glove box and magnetically stirred at 70 ^o^C for 24 h to dissolve completely to obtain 2.5 M Li_2_S_6_ and Li_2_S_8_ solution, respectively.

***Preparation of GO/DMF solution:*** A mixture comprising GO and DMF, formulated at a ratio of 17 mg of GO per mL of DMF, was placed in a round-bottom flask and subjected to continuous mechanical stirring using an ARE-310 planetary centrifugal mixer at 2000 rpm for 1 h.

***Preparation of electrolyte:*** In a glove box filled with nitrogen, combine DOL and DME in a 1:1 volume ratio. Add an appropriate quantity of LiTFSI to achieve a final concentration of 1 mol L^-1^, followed by the addition of LiNO_3_ to reach a content of 1.0 wt.%, stir thoroughly, and dissolve to obtain the electrolyte.

***Preparation of S-cathode:*** After completely dissolving 0.8 g of PVDF in 56 mL of anhydrous NMP, thoroughly grind 4.8 g of sulfur powder and 2.4 g of CNT in a mortar. Then add the mixed powder into the NMP solution and sonicate for 1 hour to obtain a preliminary mixed slurry. Move the slurry to an ARE-310 planetary centrifugal mixer and stir at 2000 rpm for 1 h to obtain a uniformly mixed high-viscosity slurry. The slurry was subsequently spread onto aluminum foil using blades of different thicknesses, dried overnight at 70 °C, and then moved to a vacuum oven at 50 °C for 8 h. The sulfur loading on the electrode substrates utilized in the electrochemical tests for this study varied from approximately 1.5 and 4 mg cm^-2^.

**S.1.4. Measurements**

***Thermal shrinkage ratio:*** Various separators were individually placed in an oven, where the temperature incrementally rose from 110 °C, with each temperature maintained for 10 minutes, to examine the changes in the size of the separators. Subsequently, **Eq. S1** is employed to determine the thermal shrinkage ratio.

$Thermal shrinkage ratio \left( \% \right)={{(A}_{0}-A_{0})}/{A_{0}\times100\%}$ (**Eq. S1**)

*A_0_* represents the initial area of the separator prior to heating, while *A_1_* indicates the residual area of the separator subsequent to heating.

***Conduct cyclic voltammetry (CV) and electrochemical impedance spectroscopy (EIS):*** CV and EIS assessments utilizing the AUTOLAB impedance analyzer. Battery performance evaluation is conducted at 30 °C utilizing the battery testing apparatus supplied by LAND Electronic Co., Ltd.

***Electrolyte uptake (EU):*** To quantitatively evaluate the efficacy of different separators, they are immersed in the electrolyte for a period of 2 hours. Subsequently, **Eq.S2** is employed to determine the electrolyte uptake (*EU*).

$EU\left( \% \right)=({W_{S}-W_{O})}/{W_{O}\times100\%}$ (**Eq. S2**)

*W_S_* represents the weight of the separator post-immersion, while *W_O_* indicates the weight of the separator prior to immersion and drying. Before measuring the weight of the submerged separators, filter paper is used to remove any excess electrolyte from their surface. To minimize experimental error, three concurrent tests are conducted, and the average is computed to determine the *EU* rate.

***Electrolyte retention (ER):*** Upon full saturation of the electrolyte, the separator is placed in a 30 °C environment, and its weight is recorded every 10 min. The *ER* is calculated using the **Eq.S3**.

$ER\left( \% \right)=({W_{S}-W_{D})}/{W_{S}\times100\%}$ (**Eq. S3**)

*W_S_* and *W_D_* denote the weight of the absorbed electrolyte separator and the weight after drying for a specified duration, respectively.

***Ionic conductivity (𝜎):*** Ion conductivity was evaluated using an AUTOLAB impedance analyzer. The procedure involved positioning a separator soaked with 20 μL of electrolyte between two spacers, configured within a CR 2032 coin cell. EIS measurements are performed after a 2 h stabilization period before testing. The testing frequency range extends from 0.1 Hz to 5 MHz. It is computed using the following equation.

$\sigma=d/{(S\times R_{0})}$ (**Eq. S4**)

where *d* represents the thickness of the separator, *S* denotes the area of the separator within the battery, and *R_o_* signifies the intercept of the EIS curve with the abscissa, reflecting the battery's ohmic.

***Galvanostatic intermittent titration technique (GITT):*** *ΔE_s_* is the evolution of the steady-state voltage, and *ΔE_τ_* represents the voltage variation during the constant-current pulse, including the IR drop. The Li-ion diffusion coefficient ($D_{{Li}^{+}}$) at various lithiation depths was calculated by fitting the collected data following the relation equation.

$D_{{Li}^{+}}=\frac{4}{\pi\tau}\frac{n_{m}^{2}V_{m}^{2}}{S}\frac{\Delta E_{S}^{2}}{\Delta E_{\tau}^{2}}$ (**Eq. S5**)

Where τ is the current pulse (s), nm is the number of moles (mol), *V_m_* is the molar volume of the active material (cm^3^ mol^-1^), and S is the electrode/electrolyte contact area (cm^2^).

**Table S1**. EA, TGA, ICP, and BET of POP, S-POP, and Pt-S-POP.

| Sample | Element Analysis  (wt.%) | | | TGA | | | ICP  (wt.%) | BET | |
| --- | --- | --- | --- | --- | --- | --- | --- | --- | --- |
|  | C | S | H | T_d5_  (^o^C) | T_d10_  (^o^C) | Yield  (wt.%) |  | Surface area  (m^2^ g^-1^) | Pore size  (nm) |
| POP | 78.4 | 4.9 | - | 532.6 | 634.4 | 83.3 | - | 194.4 | 0.4~2.9 |
| S-POP | 78.5 | 10.0 | - | 390.6 | 493.7 | 74.7 | - | 301.5 | 0.4~3.5 |
| Pt-S-POP | 72.5 | 7.4 | 6.0 | 433.8 | 555.6 | 81.6 | 6.0 | 282.4 | 0.3~3.2 |

**Table S2**. Fitting proposes a plausible coordination structure of Pt-S-POP.

| Sample | Shell | N | R (Å) | ΔE_0_ (eV) | σ^2^ (Å^2^) | R-factor (×10^-3^) |
| --- | --- | --- | --- | --- | --- | --- |
| **Pt-S-POP** | Pt-P | 1.9 ± 0.2 | 2.25 ± 0.04 | 7.9 | 0.006 | 0.6 |
|  | Pt-S | 1.9 ± 0.2 | 2.33 ± 0.03 | 2.2 | 0.002 |  |


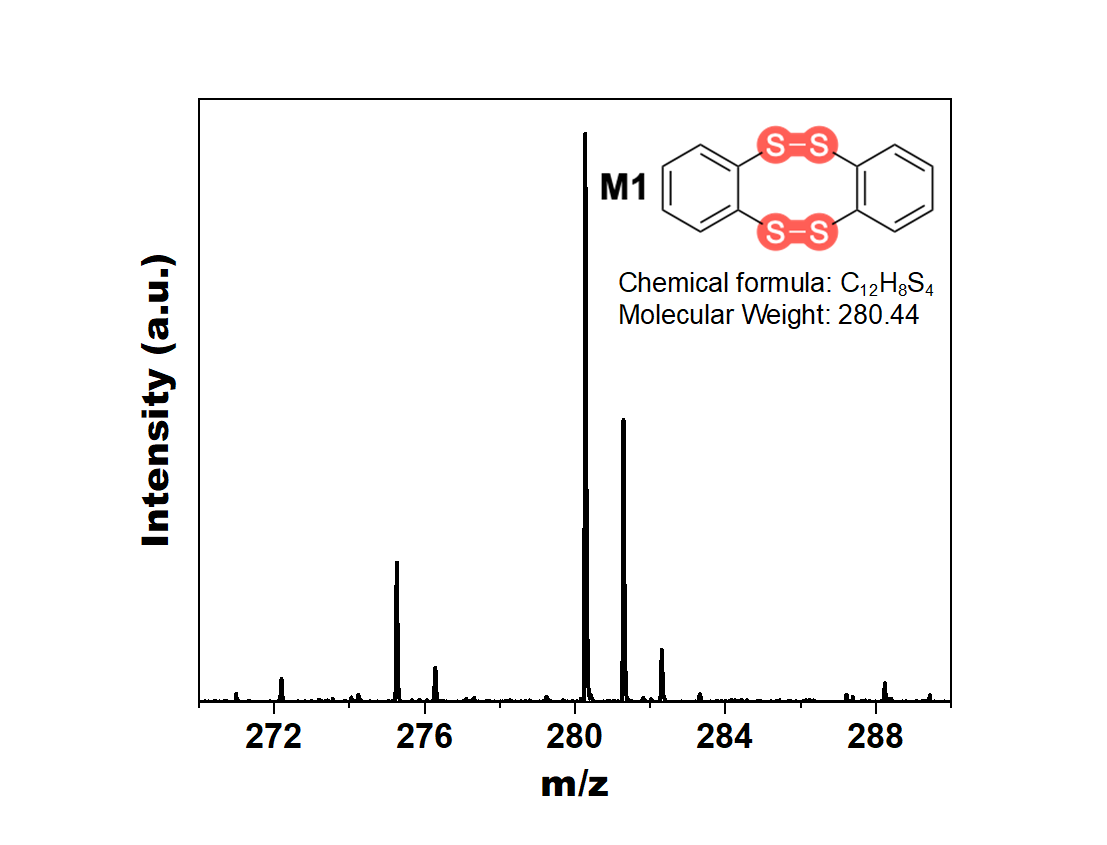


**Figure S1**. Mass spectrum of M1.


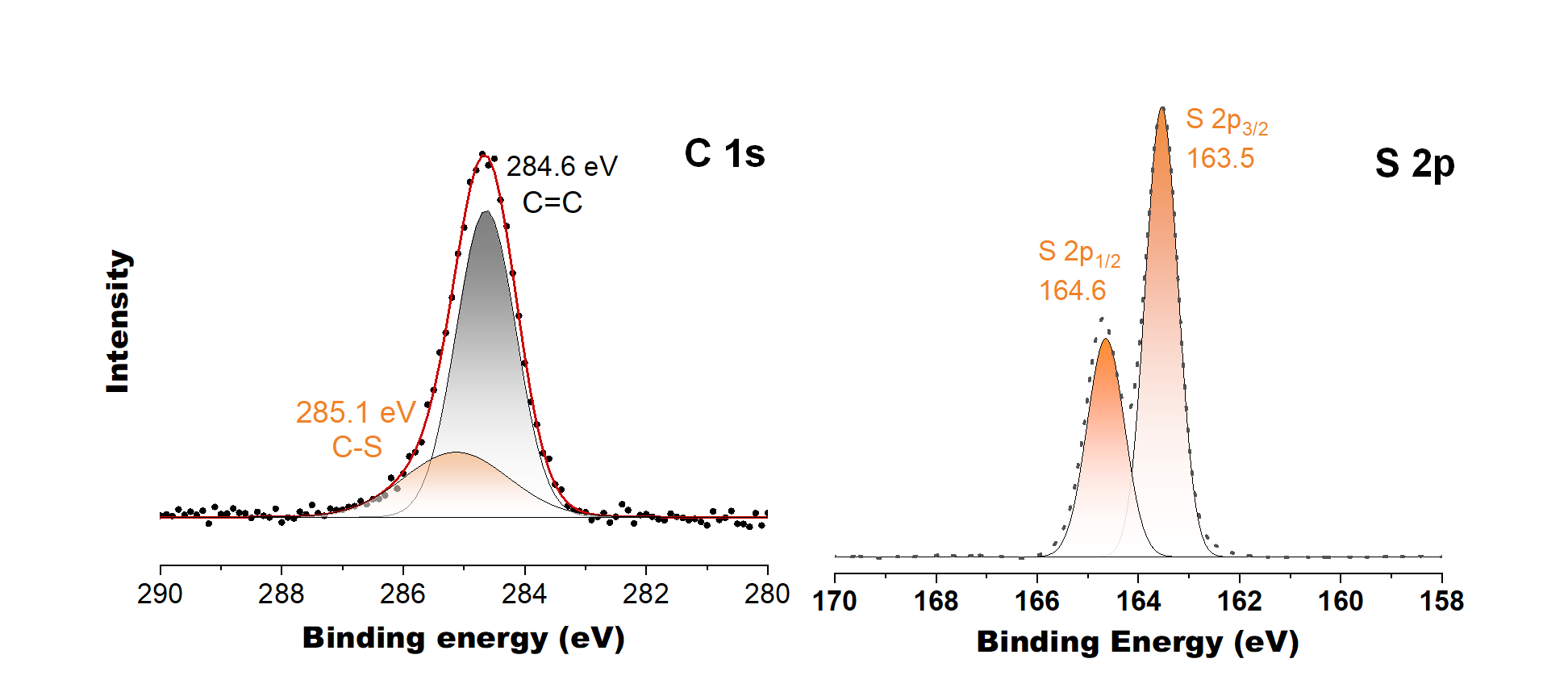


**Figure S2**. XPS analysis data of C 1s and S 2p of M1.


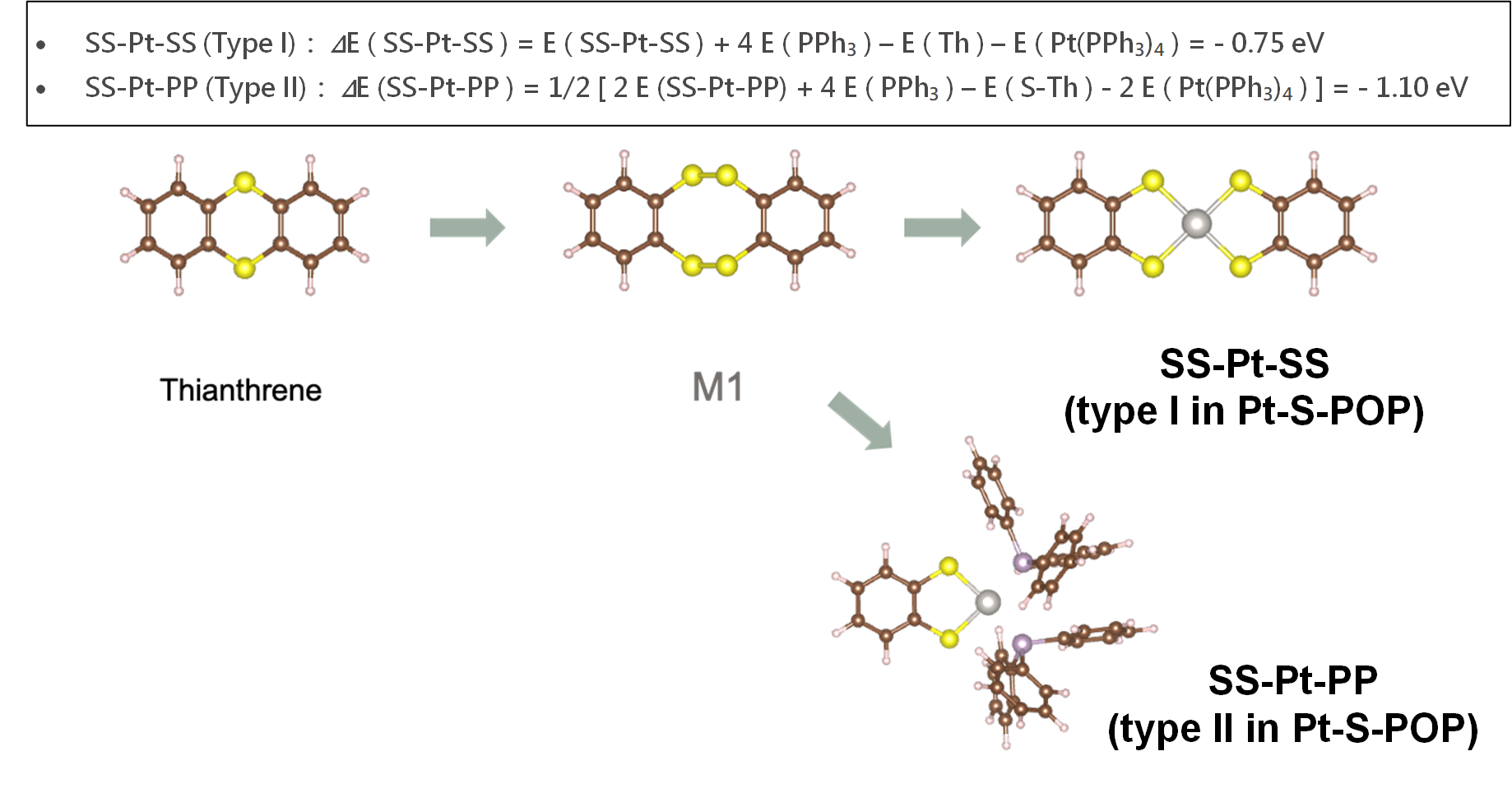


**Figure S3**. DFT-calculated reaction pathways and corresponding energy changes (*ΔE*) for the formation of single-atom Pt coordination sites within the Pt-S-POP framework.


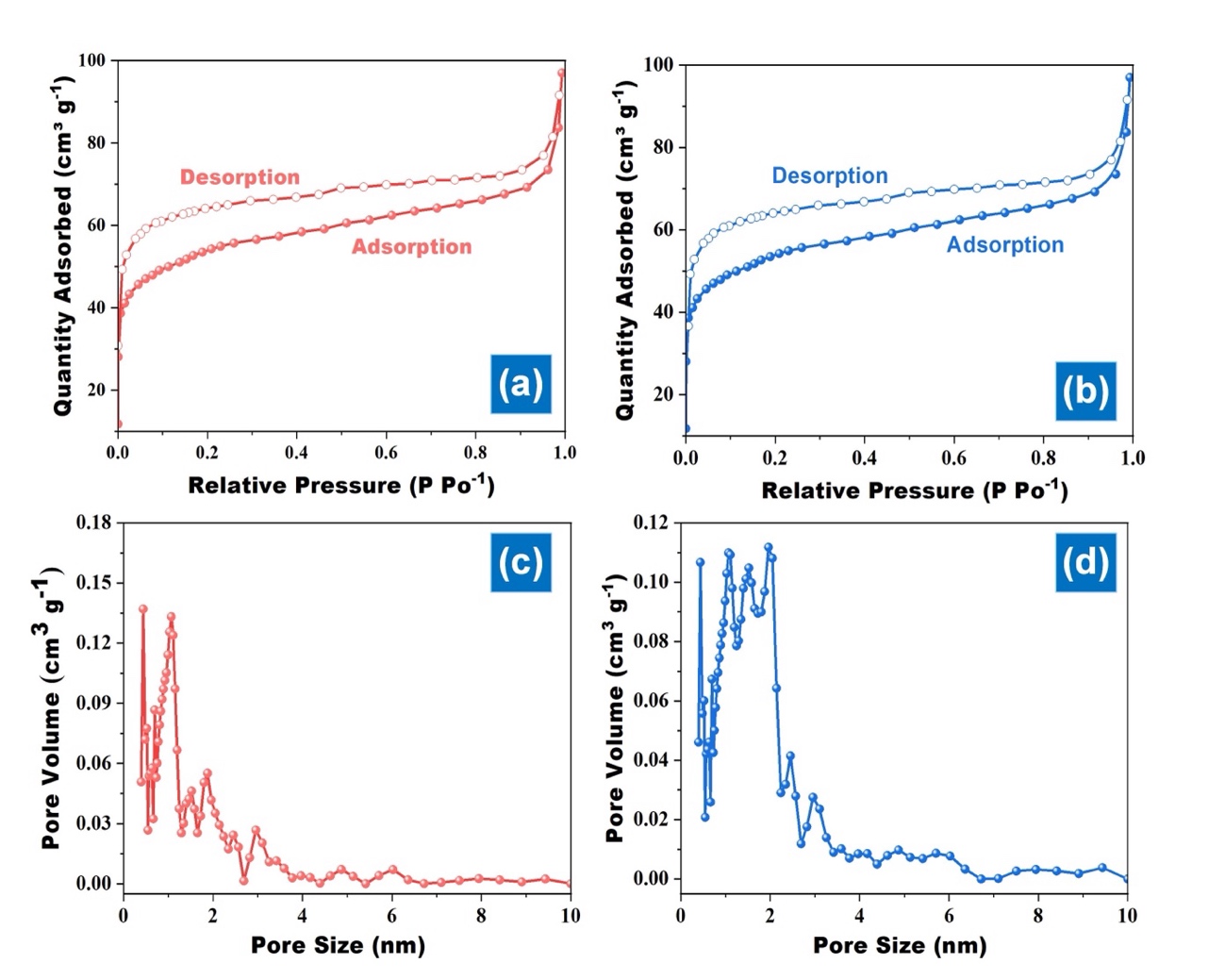


**Figure S4**. N_2_ physisorption isotherm N_2_ of (a) POP and (b) S-POP recorded at 77 K. Pore width and cumulative pore volume distribution obtained through the BJH method from the desorption branch of the N_2_ isotherm at 77 K.


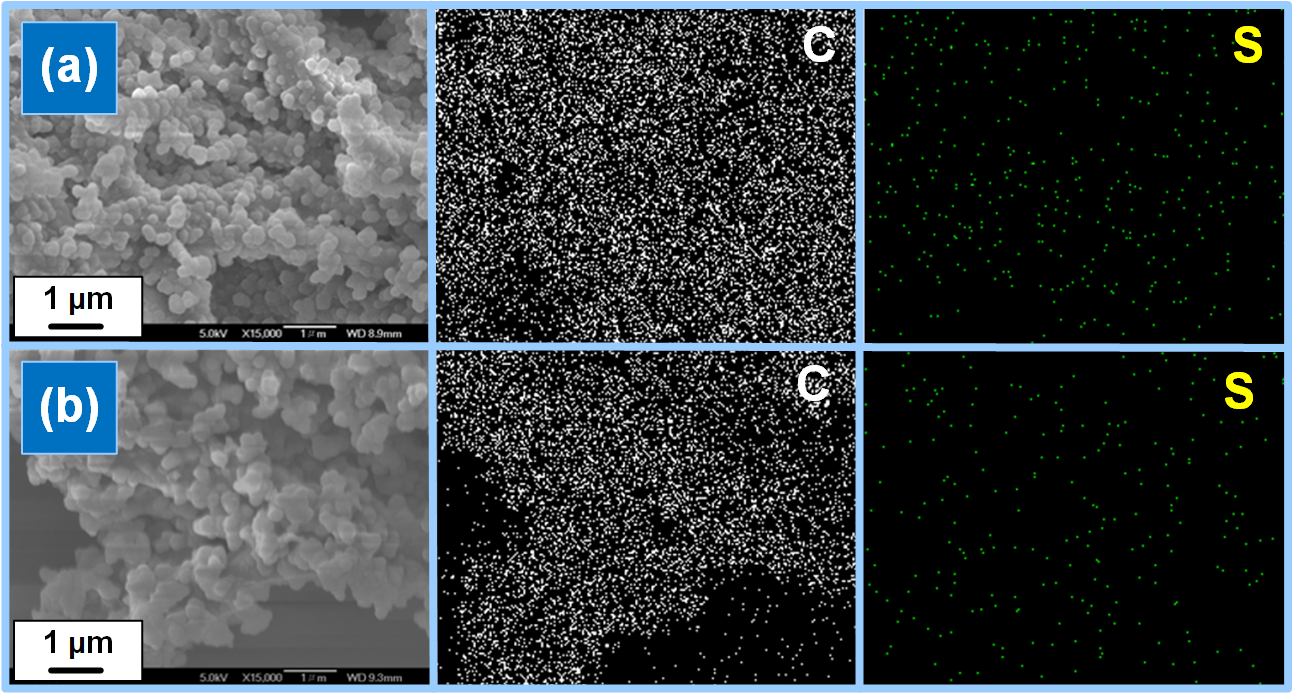


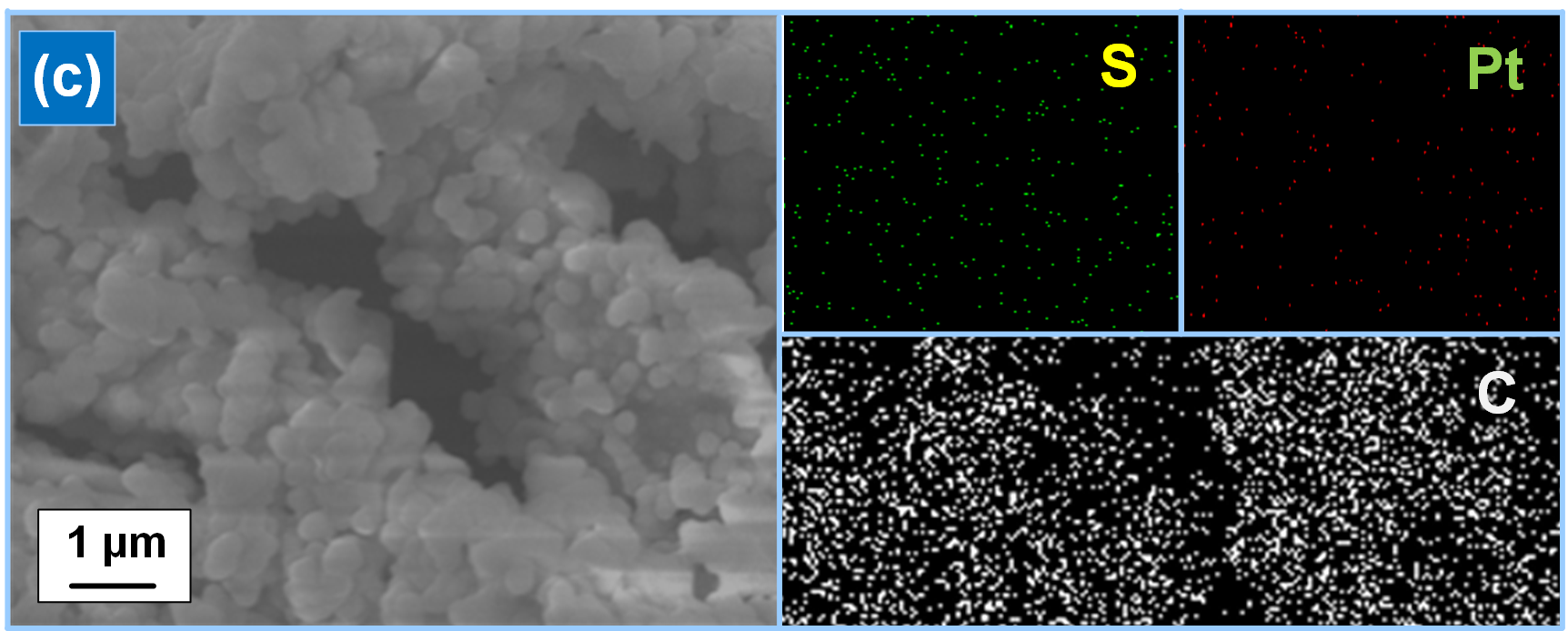


**Figure S5**. SEM images and corresponding EDX mapping images of (a) POP, (b) S-POP, and (c) Pt-S-POP.


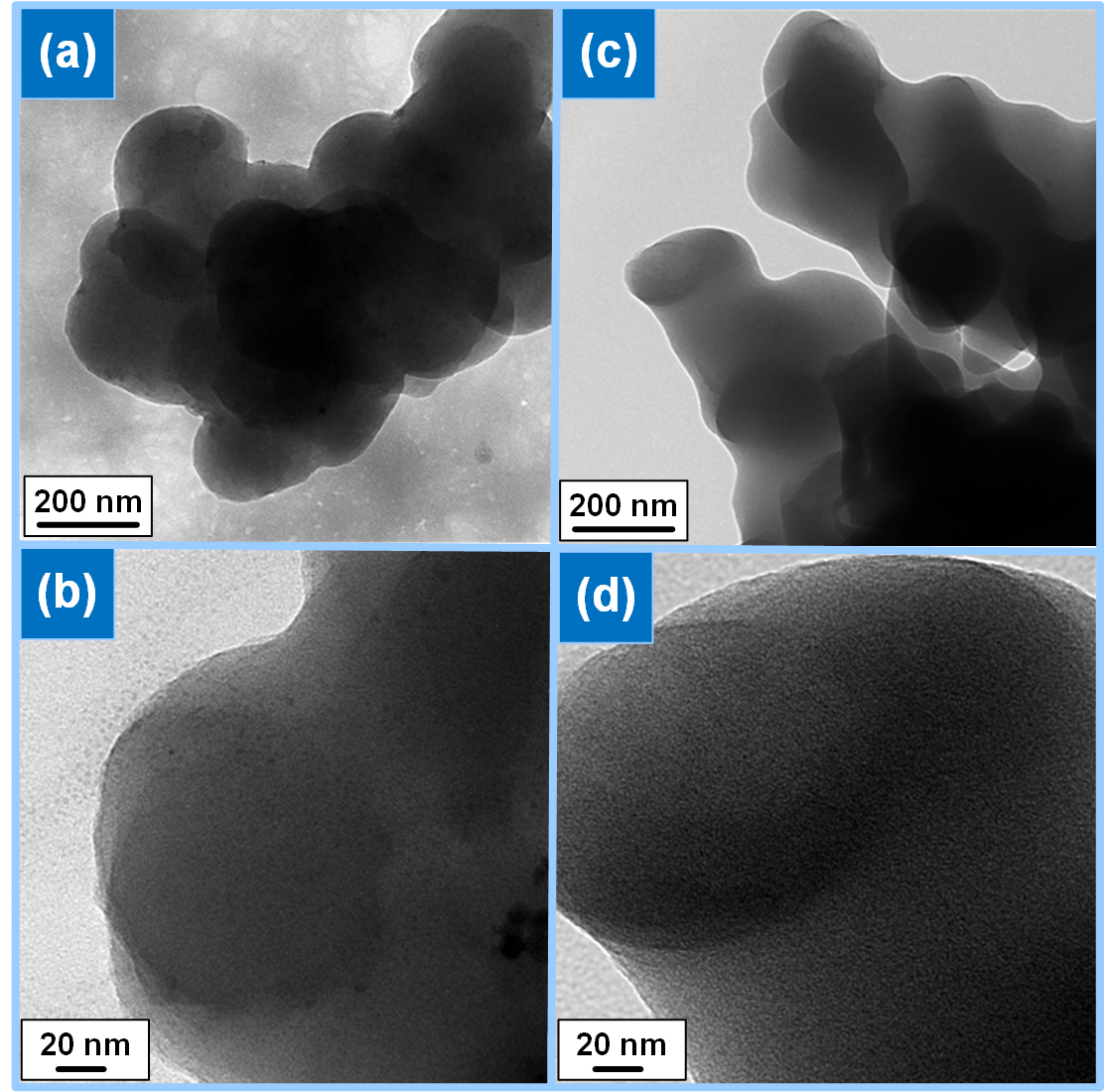


**Figure S6**. TEM images of (a, b) POP and (c, d) S-POP.


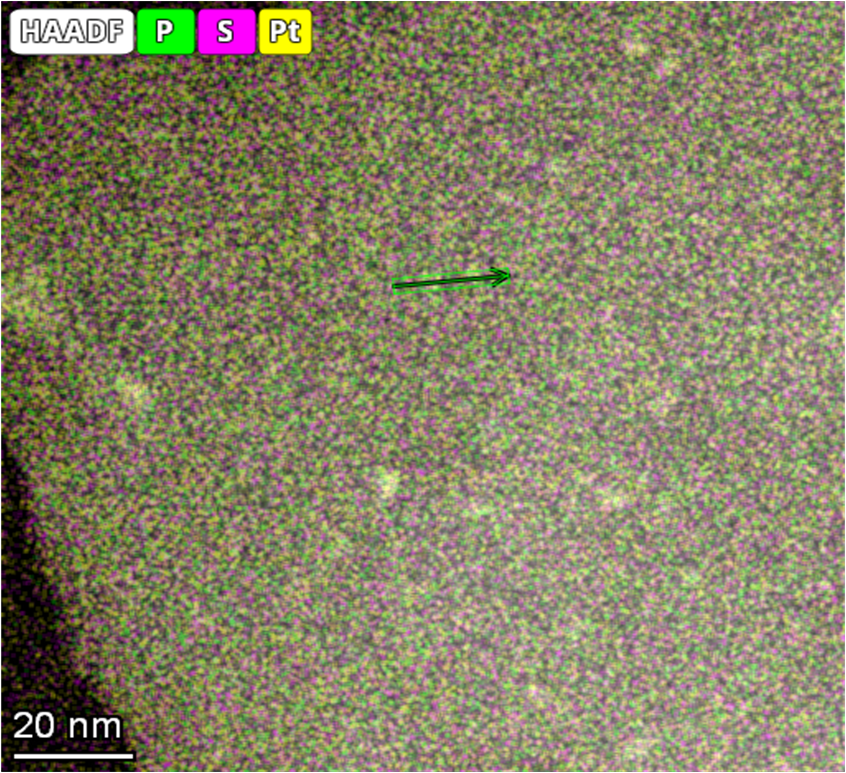


**Figure S7**. STEM-EDS elemental mapping of Pt-S-POP.


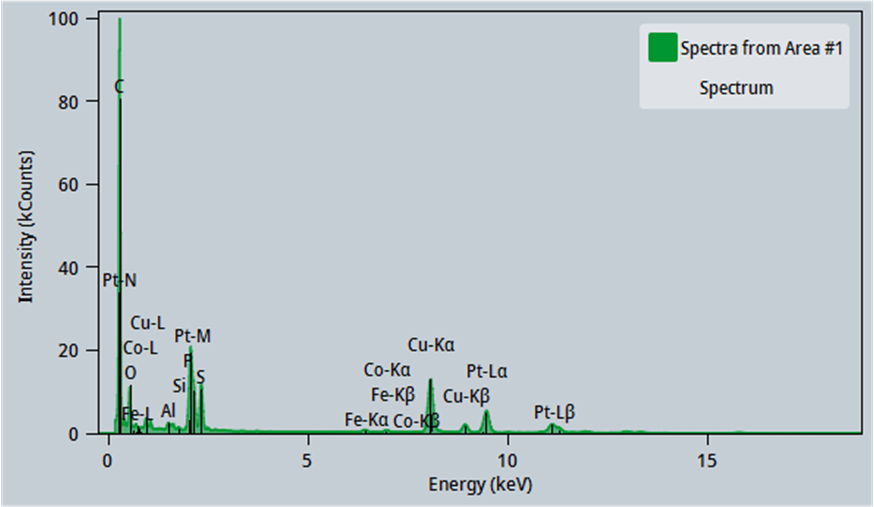


| Z | Element | Family | Atomic Fraction (%) | Atomic Error (%) | Mass Fraction (%) | Mass Error (%) | Fit Error (%) |
| --- | --- | --- | --- | --- | --- | --- | --- |
| 6 | C | K | 88.56 | 1.07 | 50.97 | 2.23 | 0.83 |
| 15 | P | K | 1.66 | 0.33 | 2.47 | 0.49 | 6 |
| 16 | S | K | 5.74 | 1.01 | 8.83 | 1.54 | 0.16 |
| 78 | Pt | L | 4.04 | 0.41 | 37.73 | 2.58 | 0.04 |

**Figure S8**. EDS, atomic and mass fraction analyses of Pt-S-POP.


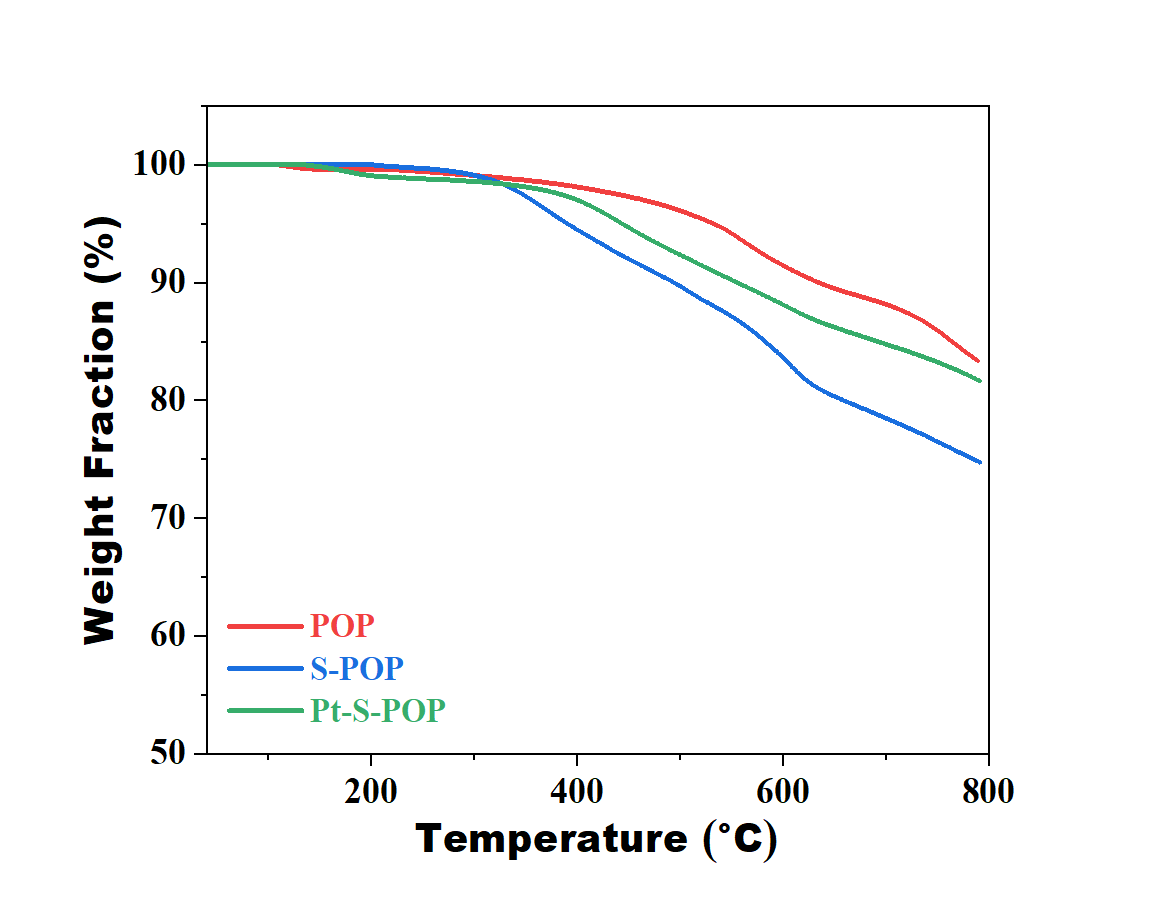


**Figure S9**. TGA curves of POP, S-POP, and Pt-S-POP.


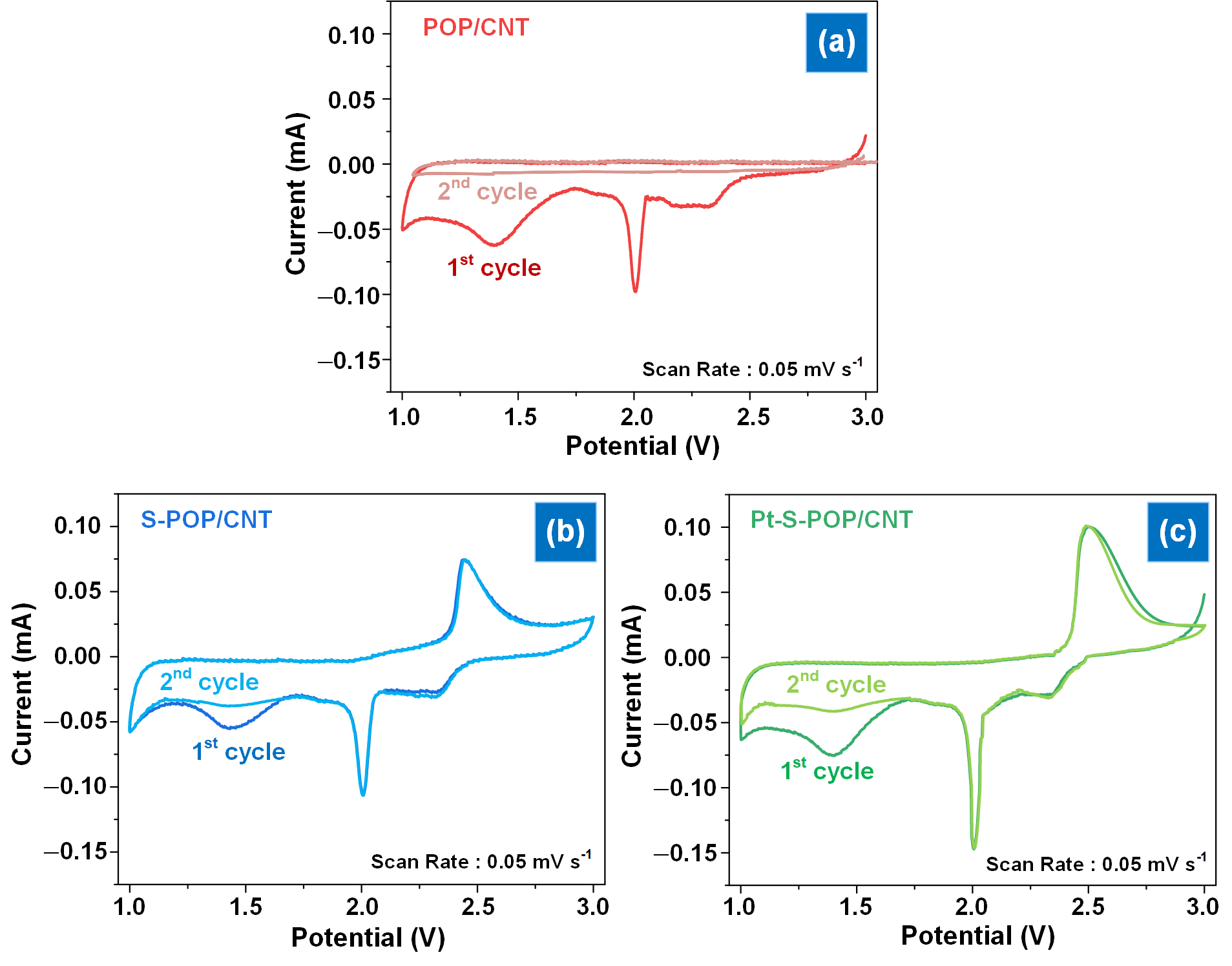


**Figure S10**. CV curves of the cell with (a) POP, (b) S-POP/CNT and (c) Pt-S-POP/CNT cathodes.


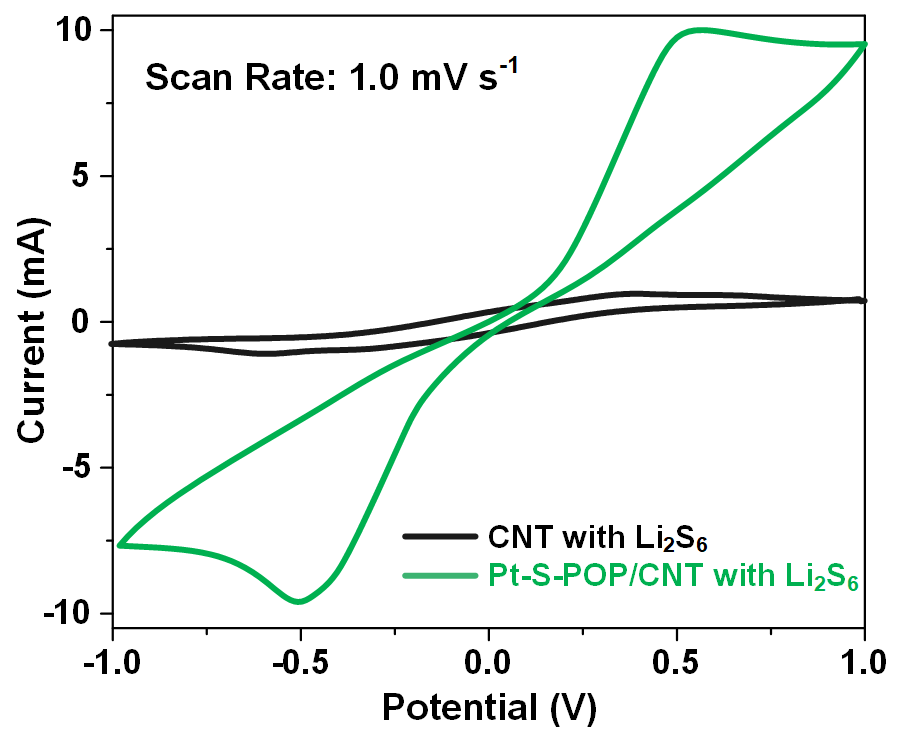


**Figure S11.** CV curves of symmetric cells with CNT and Pt-S-POP/CNT with Li_2_S_6_.


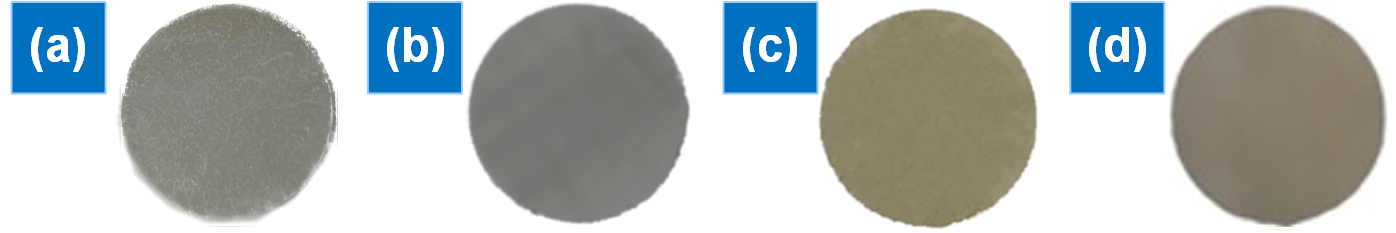


**Figure S12**. Digital photos of (a) rGO, (b) POP/rGO, (c) S-POP/rGO, and (d) Pt-S-POP/rGO separators.


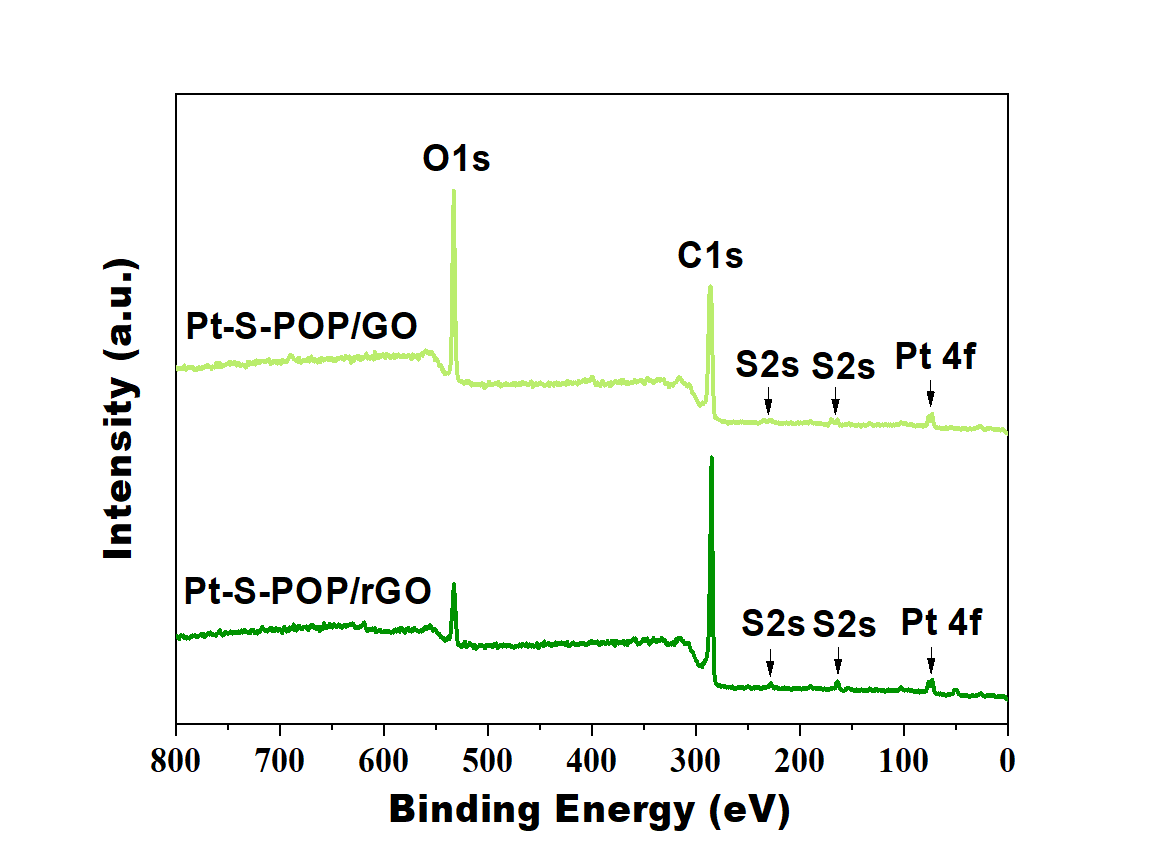


**Figure S13**. Survey XPS spectra of rGO/Pt-S-POP modified separator before and after reduction.


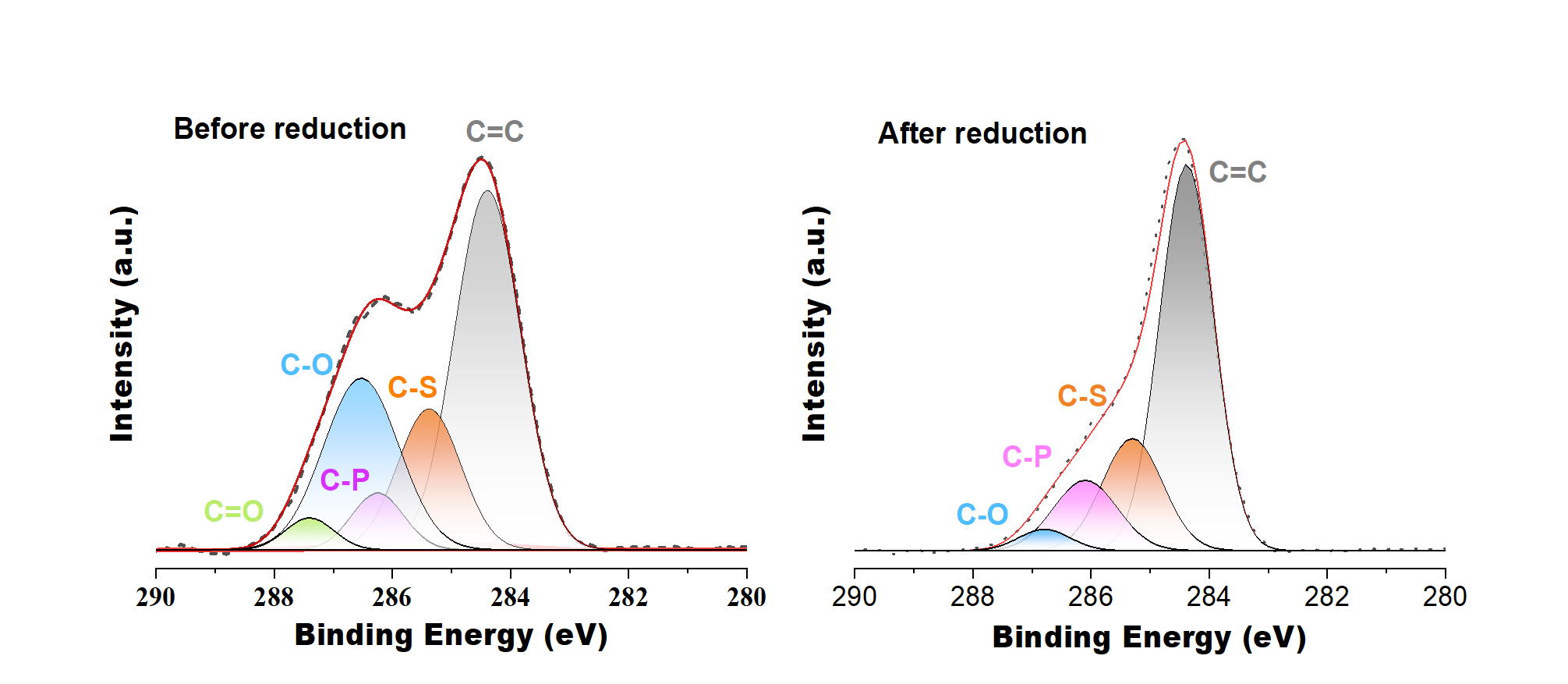


**Figure S14**. High resolution XPS spectra of the C 1s fitting for rGO/Pt-S-POP modified separators before and after reduction.


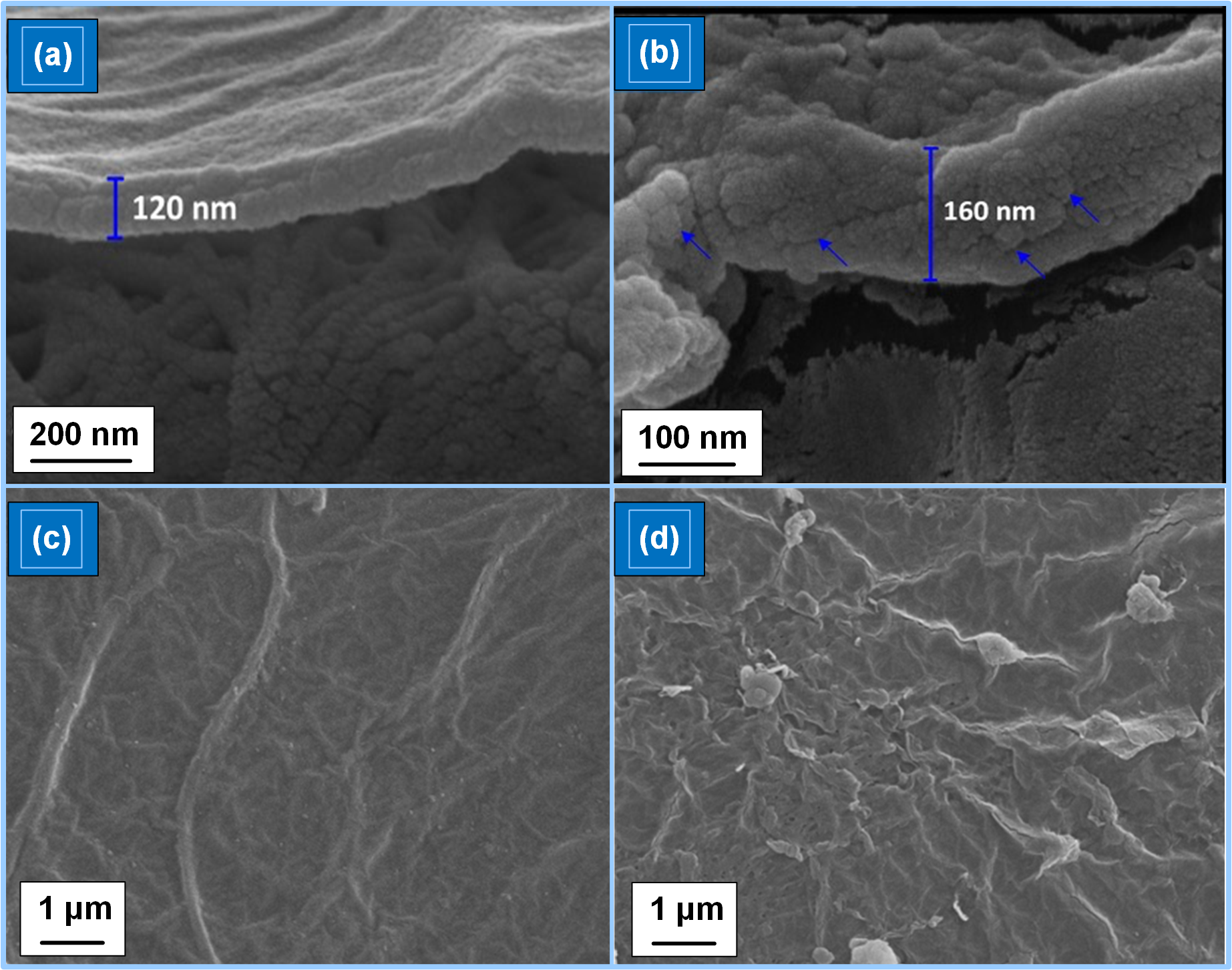


**Figure S15**. SEM images of cross-section and surface for (a, c) rGO and (b, d) rGO/Pt-S-POP separators.


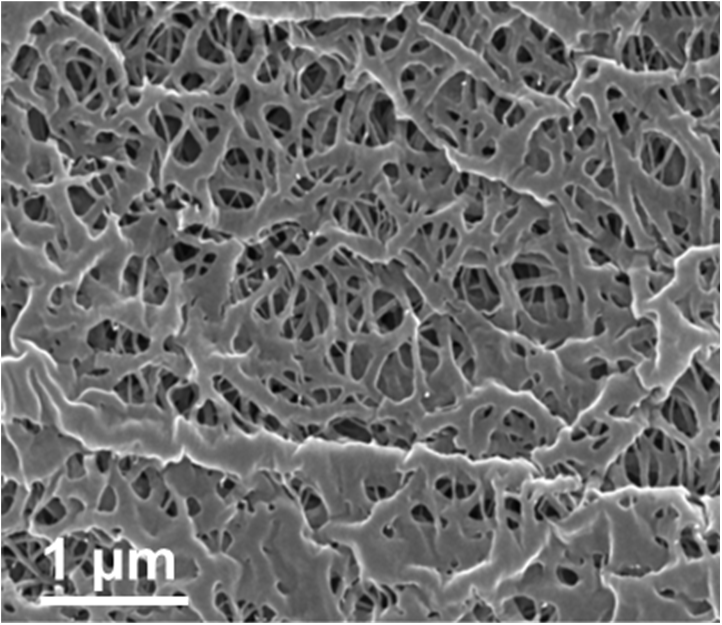


**Figure S16**. SEM image of the top view of PE separator.


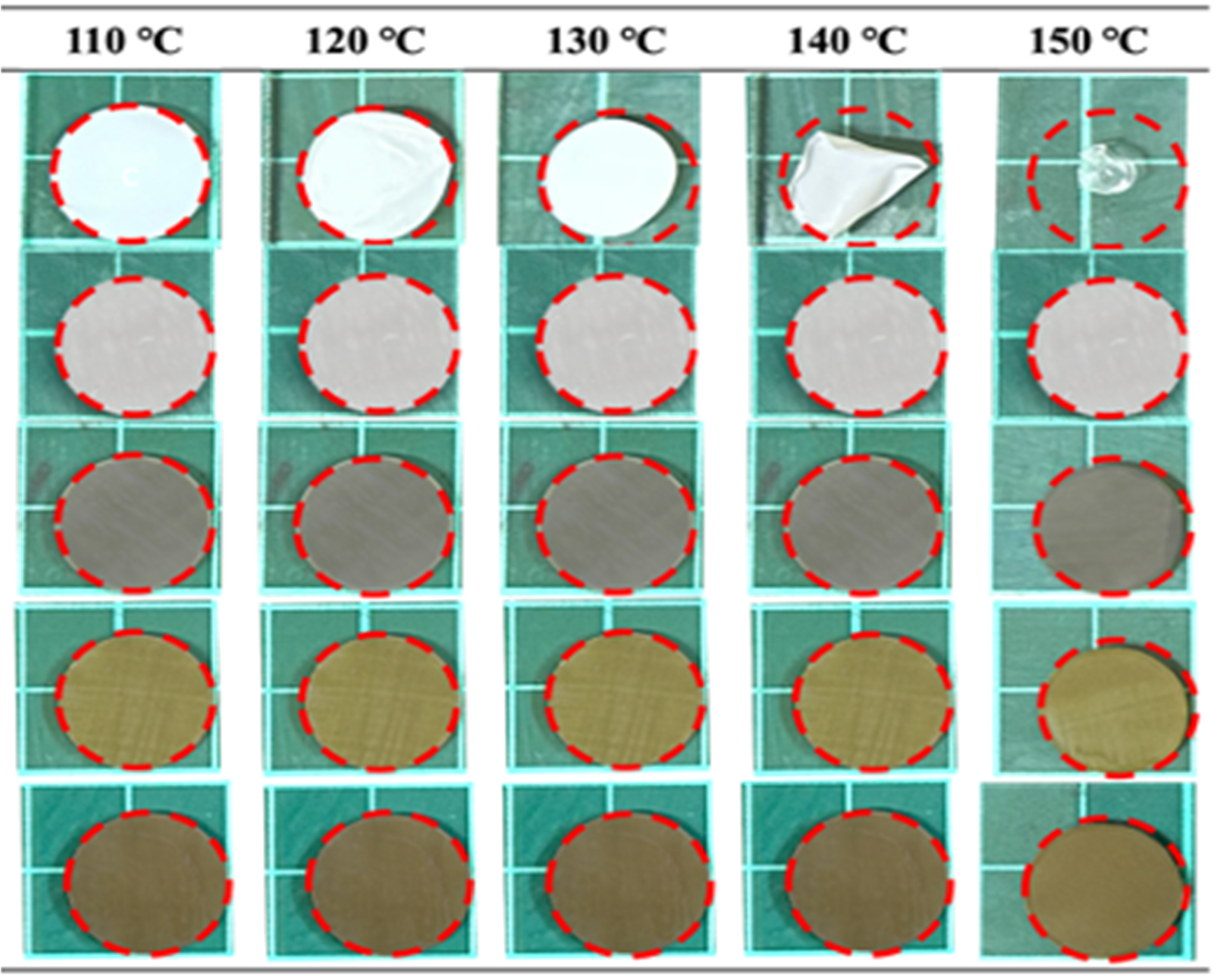


**Figure S17**. Thermal shrinkage of PE, rGO, rGO/POP, rGO/S-POP and rGO/Pt-S-POP separators at 150 ^o^C for 3 min.


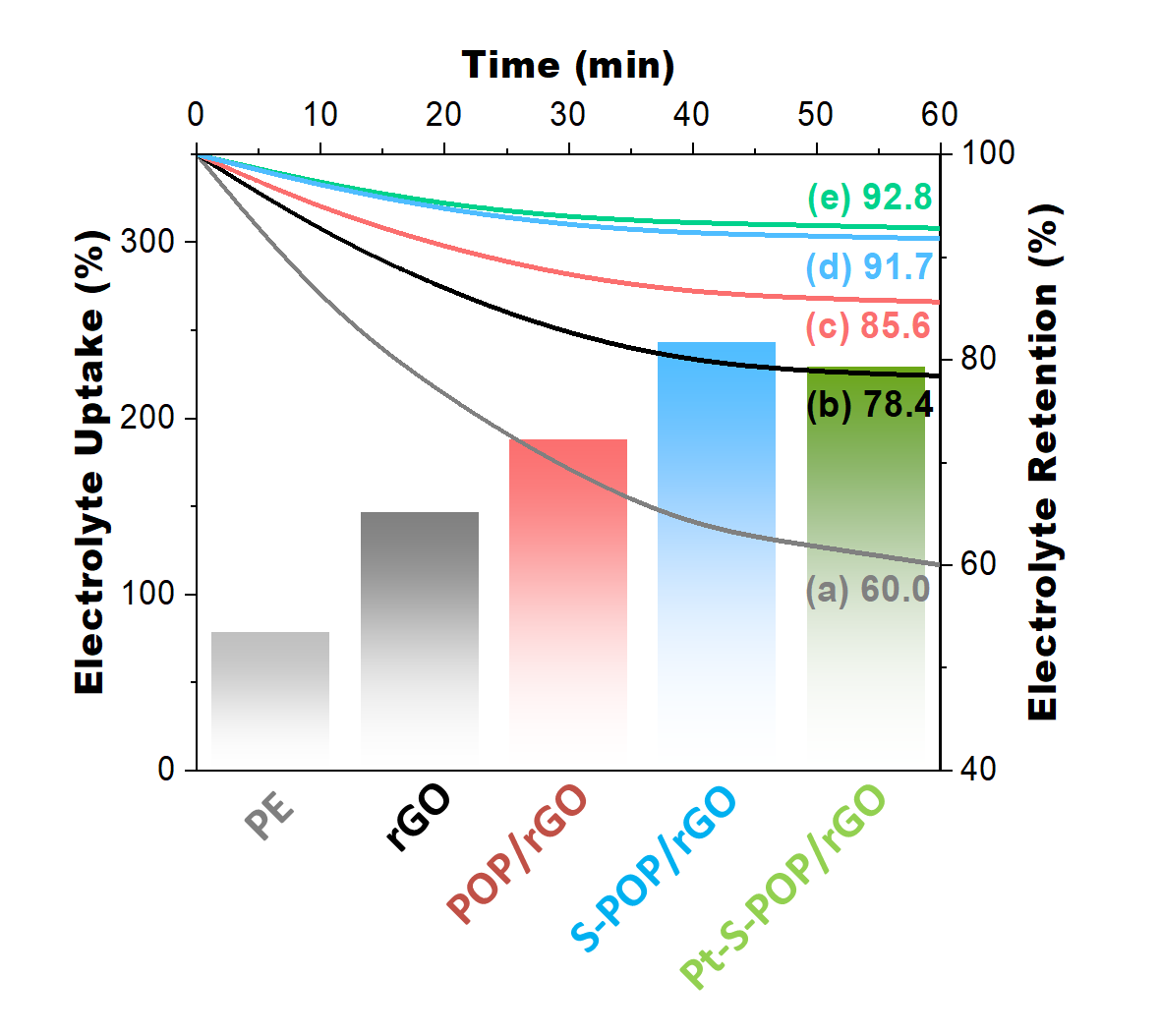


**Figure S18**. *EU* and *ER* of PE and modified separators.


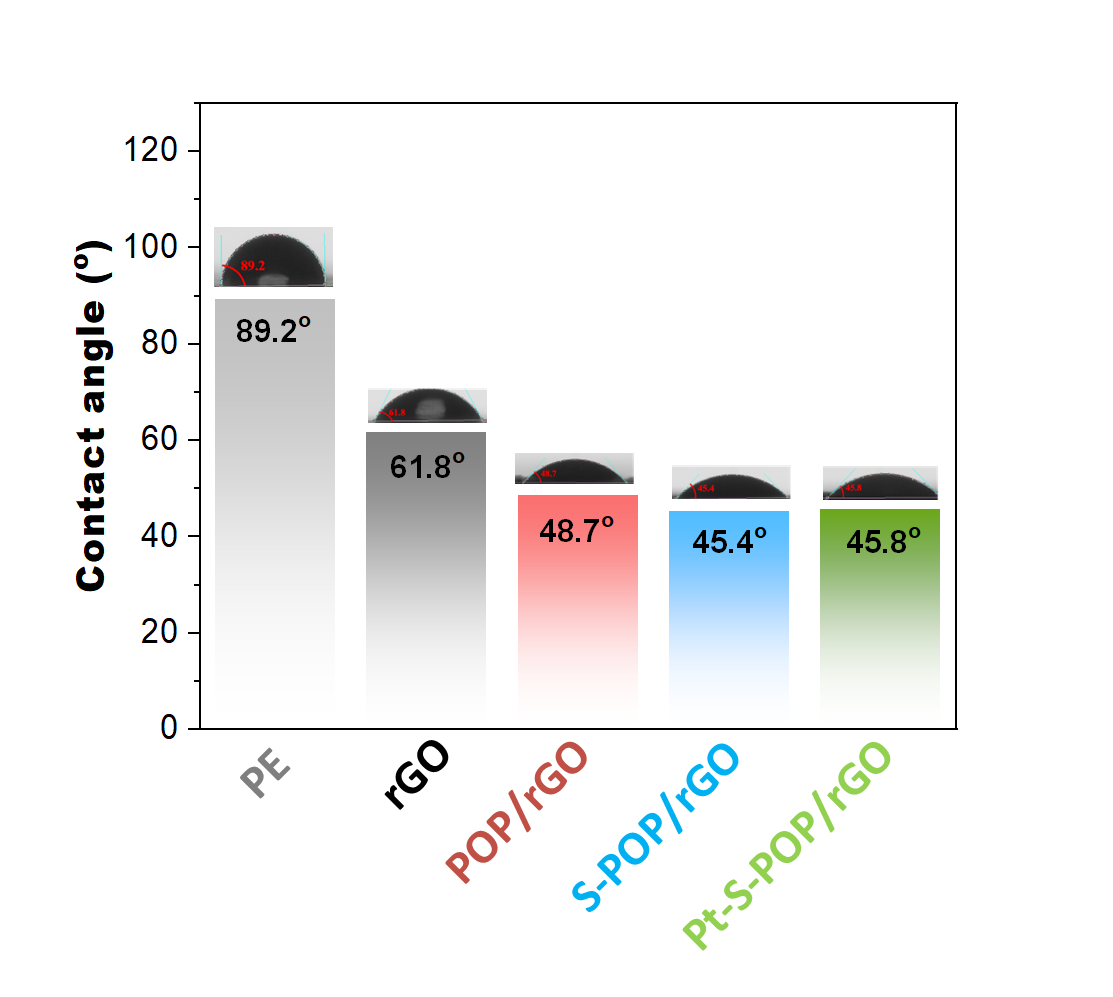


**Figure S19**. Contact angle measurements on PE and modified separators using electrolyte.


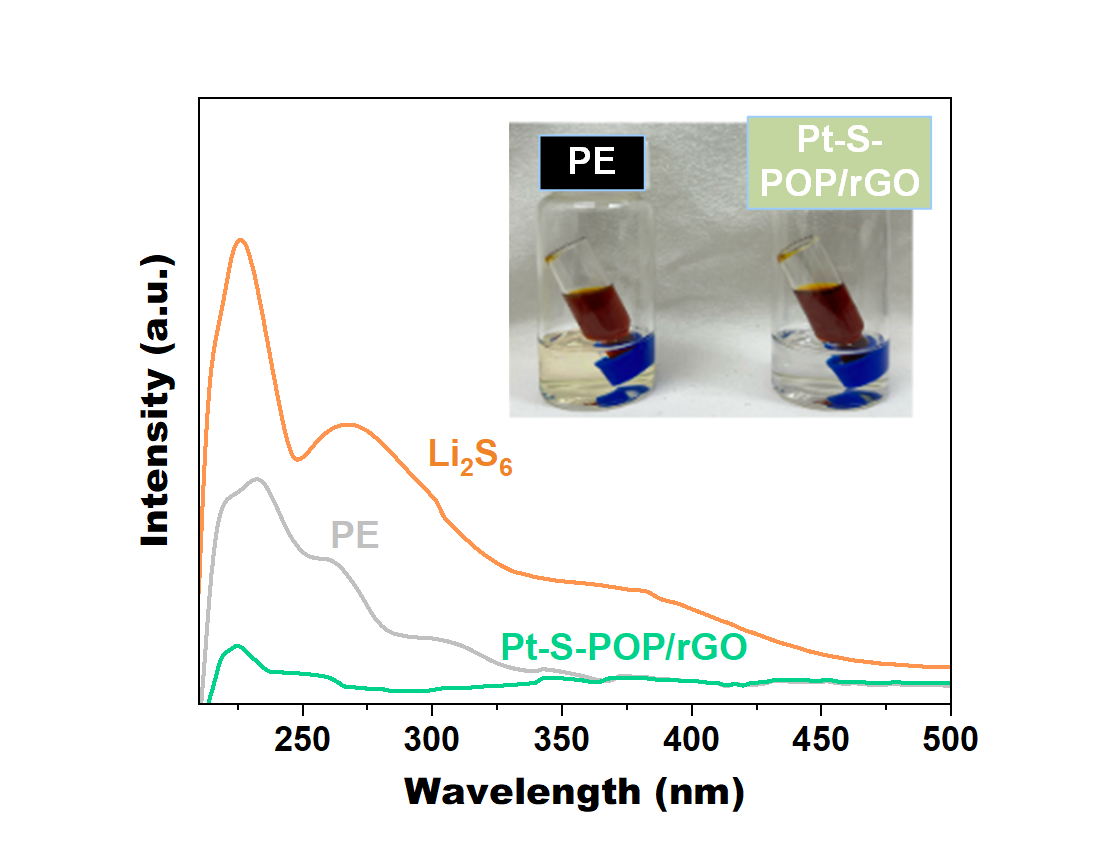


**Figure S20**. UV–vis spectra and the photographs of the Li_2_S_6_ solutions test of PE and Pt-S-POP/rGO after standing 24 h.


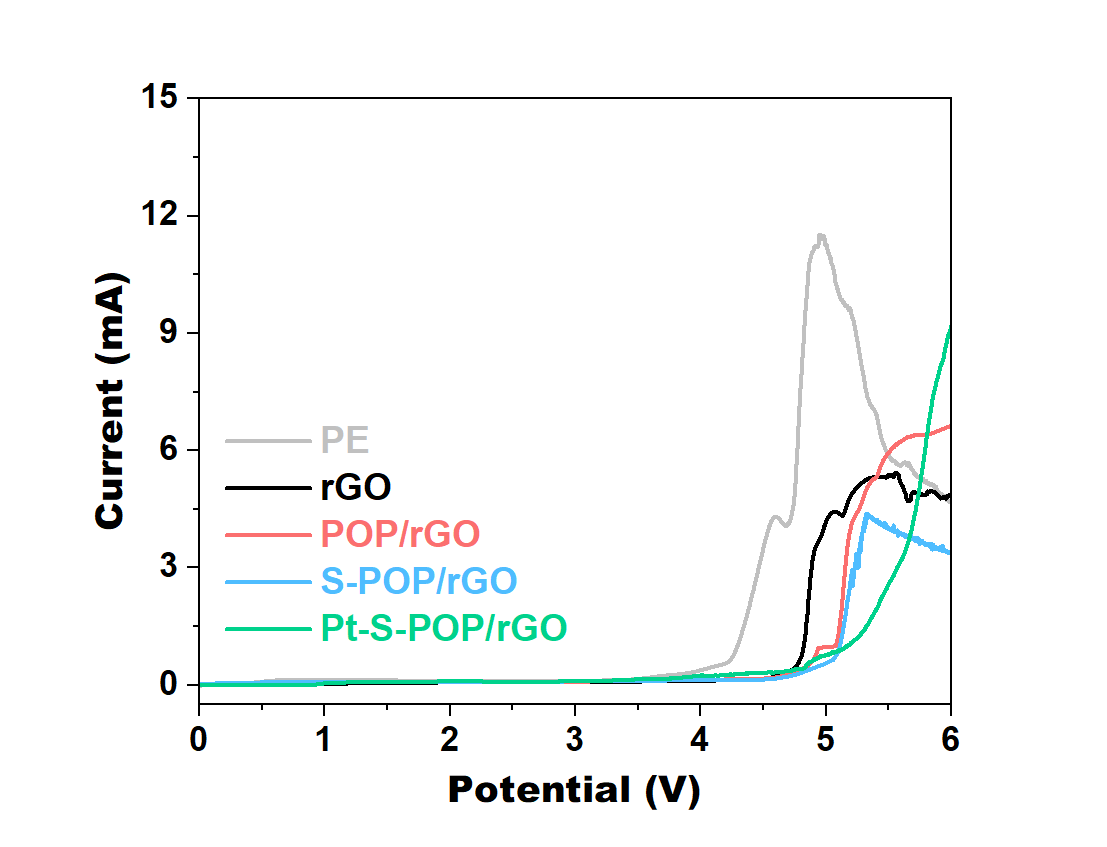


**Figure S21**. LSV curves of PE and modified separators.


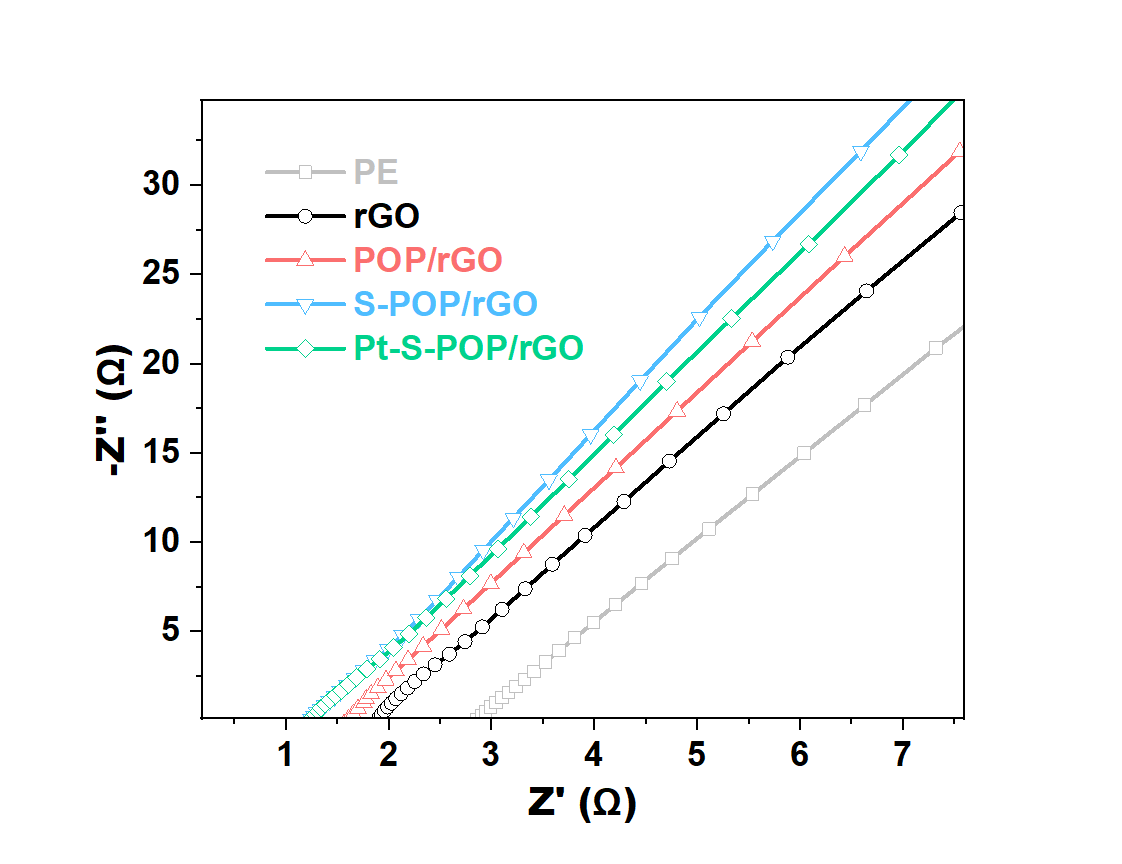


**Figure S22**. Schematic representation of Nyquist plot of different separators.


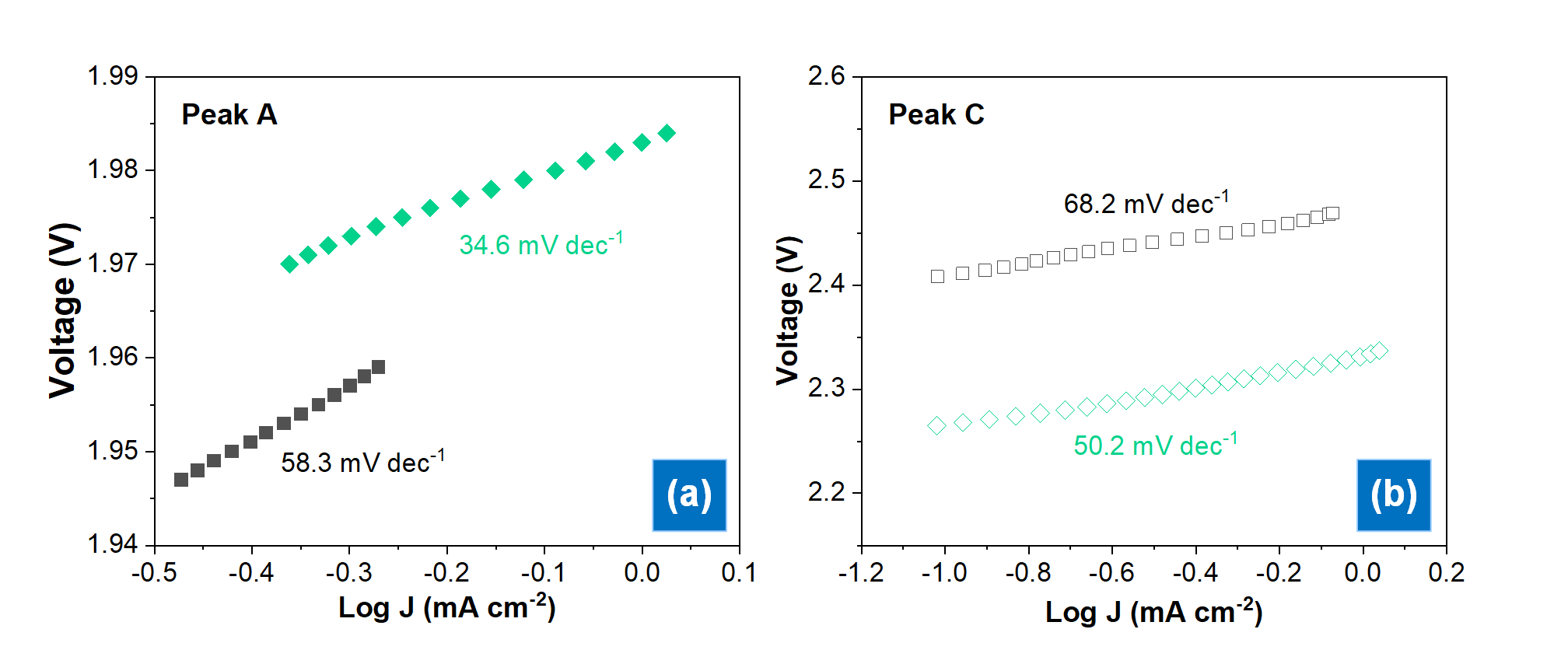


**Figure S23**. Tafel slopes from the CV peaks of the Li-S batteries with PE and Pt-S-POP/rGO separators.


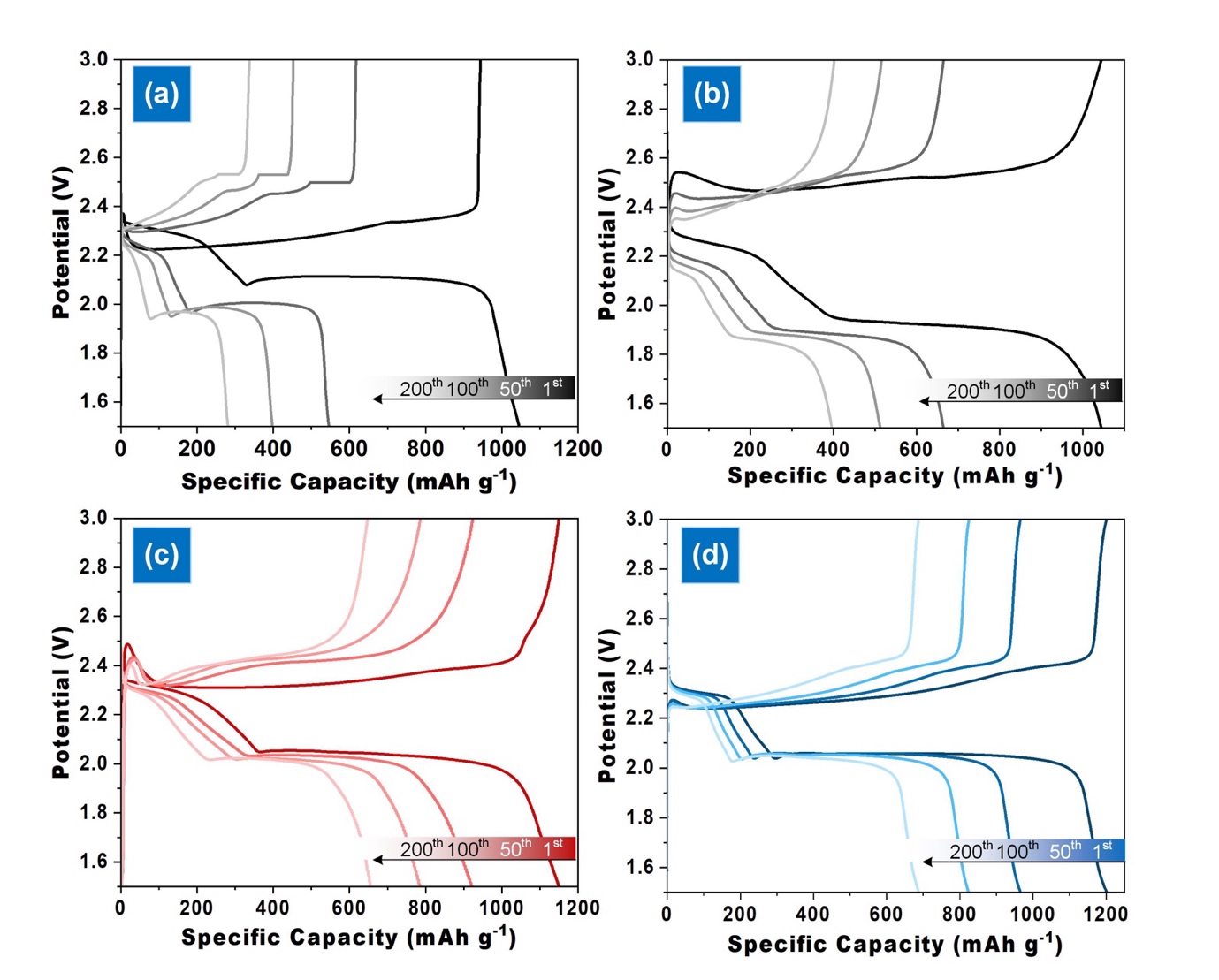


**Figure S24**. Galvanostatic discharge profiles of cell with (a) PE, (b) rGO, (c) POP/rGO, and (d) S-POP/rGO separators at 0.5 C.


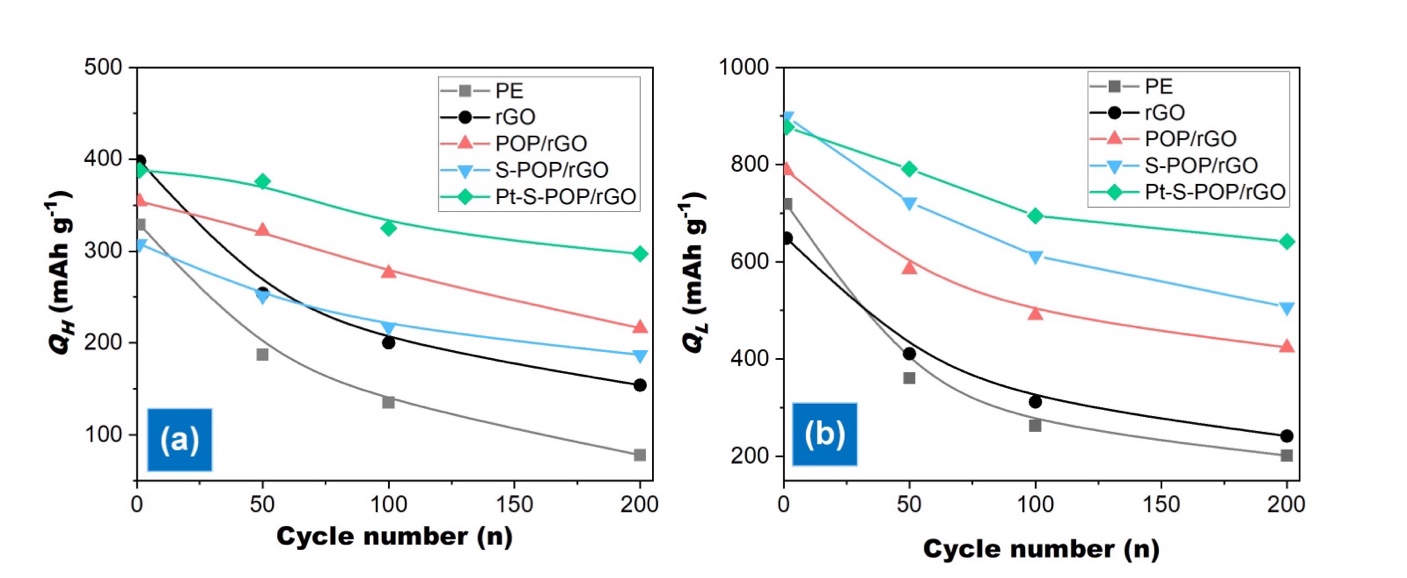


**Figure S25**. Capacity fading of the upper and lower discharge plateaus for Li-S batteries with different separators.


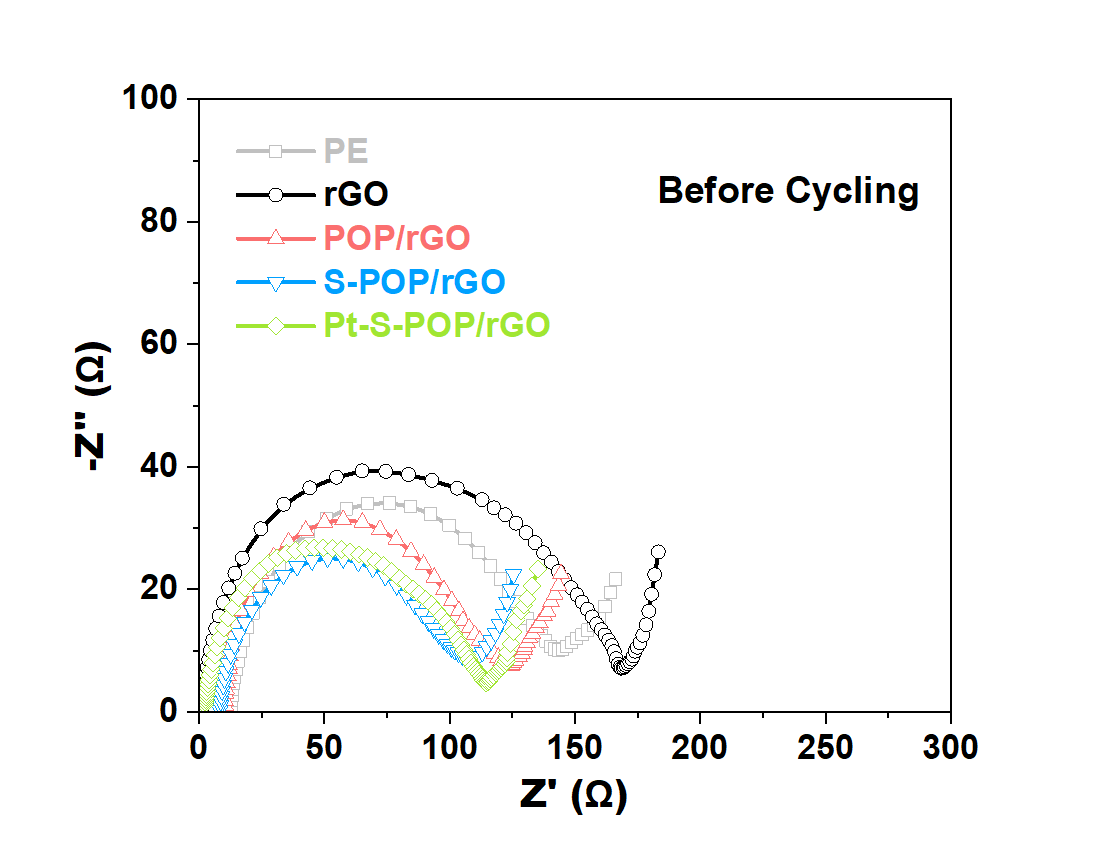


**Figure S26**. EIS of cells with different separators before cycling.


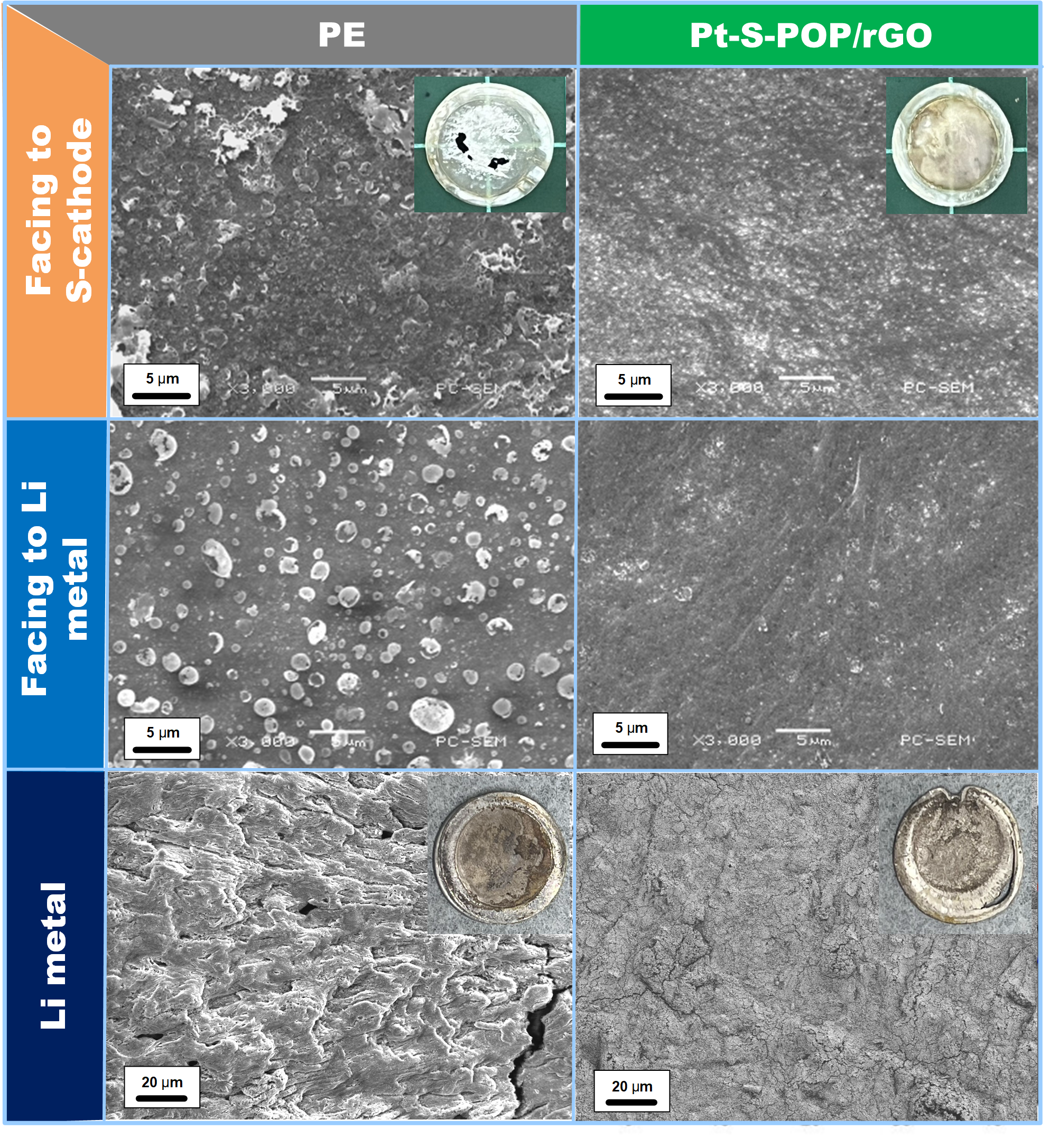


**Figure S27**. SEM images and digital photos of Li metal surface and separator surface facing Li metal for cycled cells with PE and Pt-S-POP.


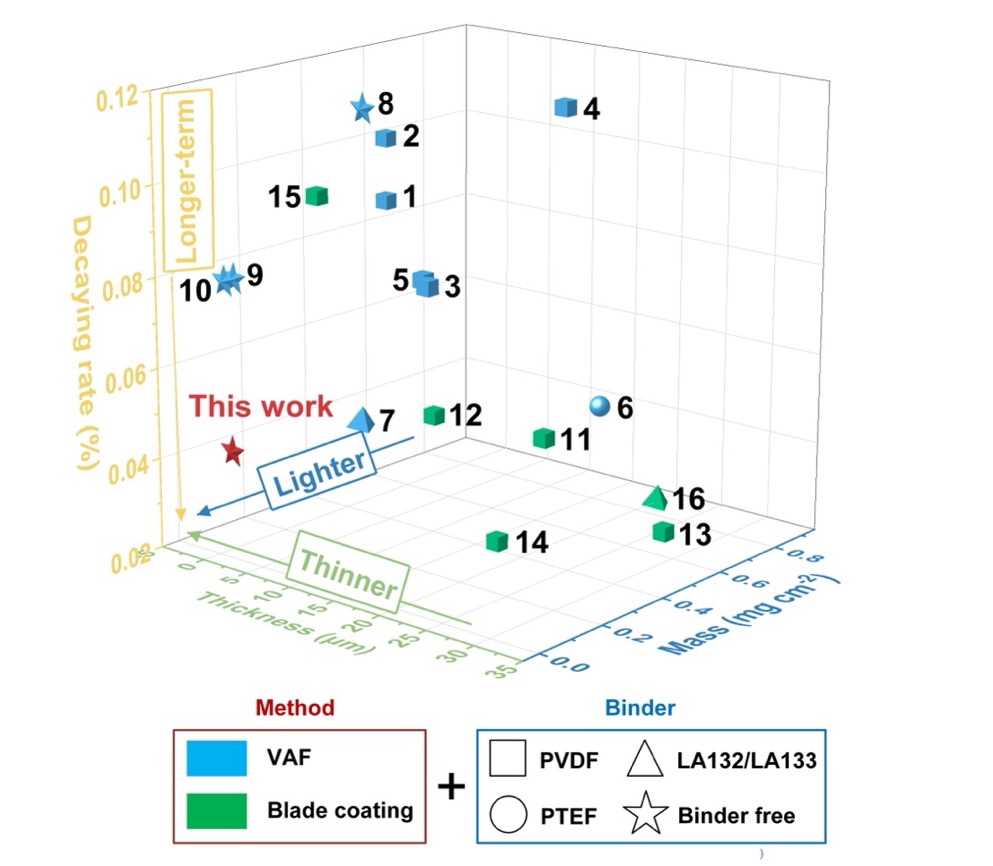


**Figure S28**. Performance of Li-S batteries with graphene-based modifiers in this study and previously reported studies.


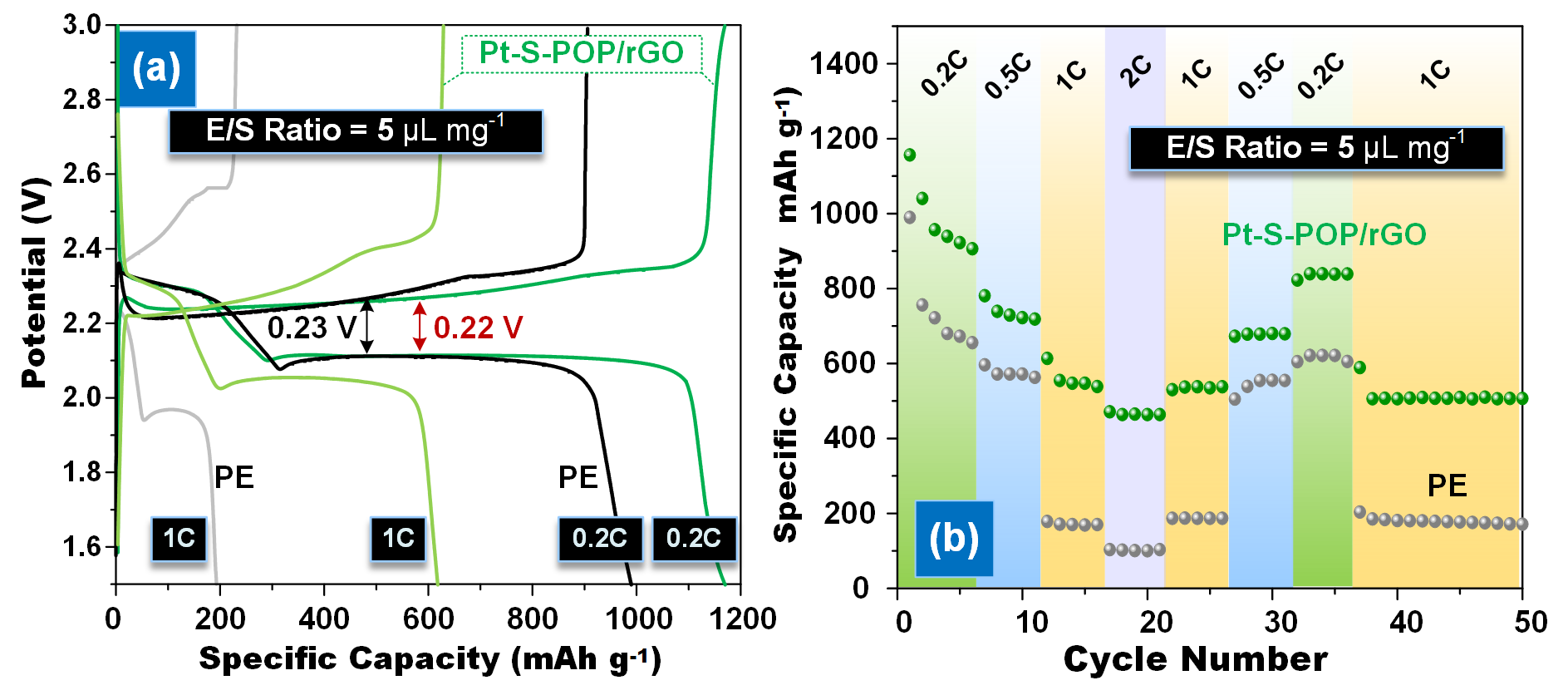


**Figure S29**. (a) Galvanostatic charge-discharge profiles and (b) rate performance of Li-S cells assembled with PE and Pt-S-POP/rGO separators under lean-electrolyte conditions (E/S = 5 μL mg⁻¹).

| **Table S3.** Comparison of separator modified with GO/or rGO in Li-S batteries.   \| **Modifier** \| **Thickness**  **/mass of modifier**  **(μm**  **/mg cm^-2^)** \| **Method** \| **binder** \| **S-loading**  **(mg cm^-2^)** \| **Initial capacity**  **(mAh g^-1^)** \| **Decaying rate**  **(%)** \| **Cycles** \| **Current**  **(C)** \| **Ref** \| \| --- \| --- \| --- \| --- \| --- \| --- \| --- \| --- \| --- \| --- \| \| **MoS_2_@CF-NrGO** \| 10/- \| Vacuum Assistant Filtration  (VAF) \| PVDF \| 1.0-1.2 \| ≈1000 \| 0.064 \| 1000 \| 1 \| [S1] \| \| **Li-MOF**  **/rGO** \| 1.2  /0.5-0.6 \| 1.2-1.4 \| ≈1480^a^ \| 0.089 \| 600 \| 1 \| [S2] \| \| **MOF/rGO** \| ≈1280^a^ \| 0.103 \| \| **Nb_2_O_5_/rGO** \| 20  /0.1-0.5 \| 1.5 \| ≈1100 \| 0.086 \| 500 \| ≈0.3 \| [S3] \| \| **CeO_2_@G** \| 25/0.38 \| 1.2 \| 1039 \| 0.12 \| 200 \| 0.5 \| [S4] \| \| **FM@G/MoS_2_** \| 12.5/0.3 \| Li_2_S_6_ solution  (0.5 M) \| 1040 \| 0.08 \| 300 \| 1 \| [S5] \| \| **rGO/MoS_2_/C** \| -/- \| 1.5 \| ~1400^a^ \| 0.002 \| 1000 \| 2 \| [S6] \| \| **SrF_2_/graphene** \| 22/0.6 \| PTFE \| - \| 1140 \| 0.05 \| 350 \| 0.5 \| [S7] \| \| **WN_0.67_@NG** \| 5.6/0.3 \| LA132 \| 1.2-1.5 \| ≈900 \| 0.045 \| 800 \| 1 \| [S8] \| \| **Ni_3_Sn_2_/NG** \| -/0.4 \| LA133 \| 1.6 \| 1022 \| 0.07 \| 400 \| 1 \| [S9] \| \| **rGO@MoS_2_** \| ≈8/0.24 \| NO \| 1.8-2.0 \| 877 \| 0.116 \| 500 \| 1 \| [S10] \| \| **Nb_2_O_5_-rGO** \| 0.2/0.05 \| 1 \| ~680^a^ \| 0.08 \| 500 \| 3 \| [S11] \| \| **CoPc@GO** \| 0.2/0.022 \| 2.5 \| 1092 \| 0.08 \| 400 \| 1 \| [S12] \| \| **PNCG** \| 24/0.35 \| Blade Coating \| PVDF \| Li_2_S_6_ solution  (0.25 M) \| 1192 \| 0.05 \| 800 \| 0.1 \| [S13] \| \| **CaF_2_@rGO** \| 14/- \| - \| 1005 \| 0.06 \| 420 \| 0.5 \| [S14] \| \| **W/NG** \| 13/0.32 \| 1.1 \| 1100 \| 0.05 \| 100 \| 2 \| [S15] \| \| **Sb_2_Se_3_/rGO** \| 32/0.5 \| 1.8 \| 945 \| 0.03 \| 500 \| 1 \| [S16] \| \| **Ni_3_B@rGO** \| 23/~0.23 \| 1.5 \| 572 \| 0.06 \| 500 \| 2 \| [S17] \| \| **Co-3DC-rGO** \| 20/- \| 1.5 \| ~1300^a^ \| 0.12 \| 500 \| 1 \| [S18] \| \| **ZnS-RGA** \| 8/0.1 \| 1.5 \| 800 \| 0.1 \| 500 \| 1 \| [S19] \| \| **Ni@C/G** \| -/≈0.4 \| 2.0 \| 1337.4 \| 0.061 \| 1000 \| 0.5 \| [S20] \| \| **LiNiPO_4_/rGO** \| 20/0.88 \| LA133 \| 1.5 \| 945 \| 0.02 \| 1400 \| 1.5 \| [S21] \| \| **Pt-S-POP/rGO** \| ~0.12/0.04 \| No \| 1.5 \|  \| 0.042 \| 1000 \| 1 \| This work \| \| 0.030 \| 2000 \|   ^a^ Value determined by reading of the data in the article. |
| --- | --- | --- | --- | --- | --- | --- | --- | --- | --- | --- | --- | --- | --- | --- | --- | --- | --- | --- | --- | --- | --- | --- | --- | --- | --- | --- | --- | --- | --- | --- | --- | --- | --- | --- | --- | --- | --- | --- | --- | --- | --- | --- | --- | --- | --- | --- | --- | --- | --- | --- | --- | --- | --- | --- | --- | --- | --- | --- | --- | --- | --- | --- | --- | --- | --- | --- | --- | --- | --- | --- | --- | --- | --- | --- | --- | --- | --- | --- | --- | --- | --- | --- | --- | --- | --- | --- | --- | --- | --- | --- | --- | --- | --- | --- | --- | --- | --- | --- | --- | --- | --- | --- | --- | --- | --- | --- | --- | --- | --- | --- | --- | --- | --- | --- | --- | --- | --- | --- | --- | --- | --- | --- | --- | --- | --- | --- | --- | --- | --- | --- | --- | --- | --- | --- | --- | --- | --- | --- | --- | --- | --- | --- | --- | --- | --- | --- | --- | --- | --- | --- | --- | --- | --- | --- | --- | --- | --- | --- | --- | --- | --- | --- | --- | --- | --- | --- | --- | --- | --- | --- | --- | --- | --- | --- | --- | --- | --- | --- | --- | --- | --- | --- | --- | --- | --- | --- | --- | --- | --- | --- | --- | --- | --- | --- | --- | --- | --- | --- | --- | --- | --- |


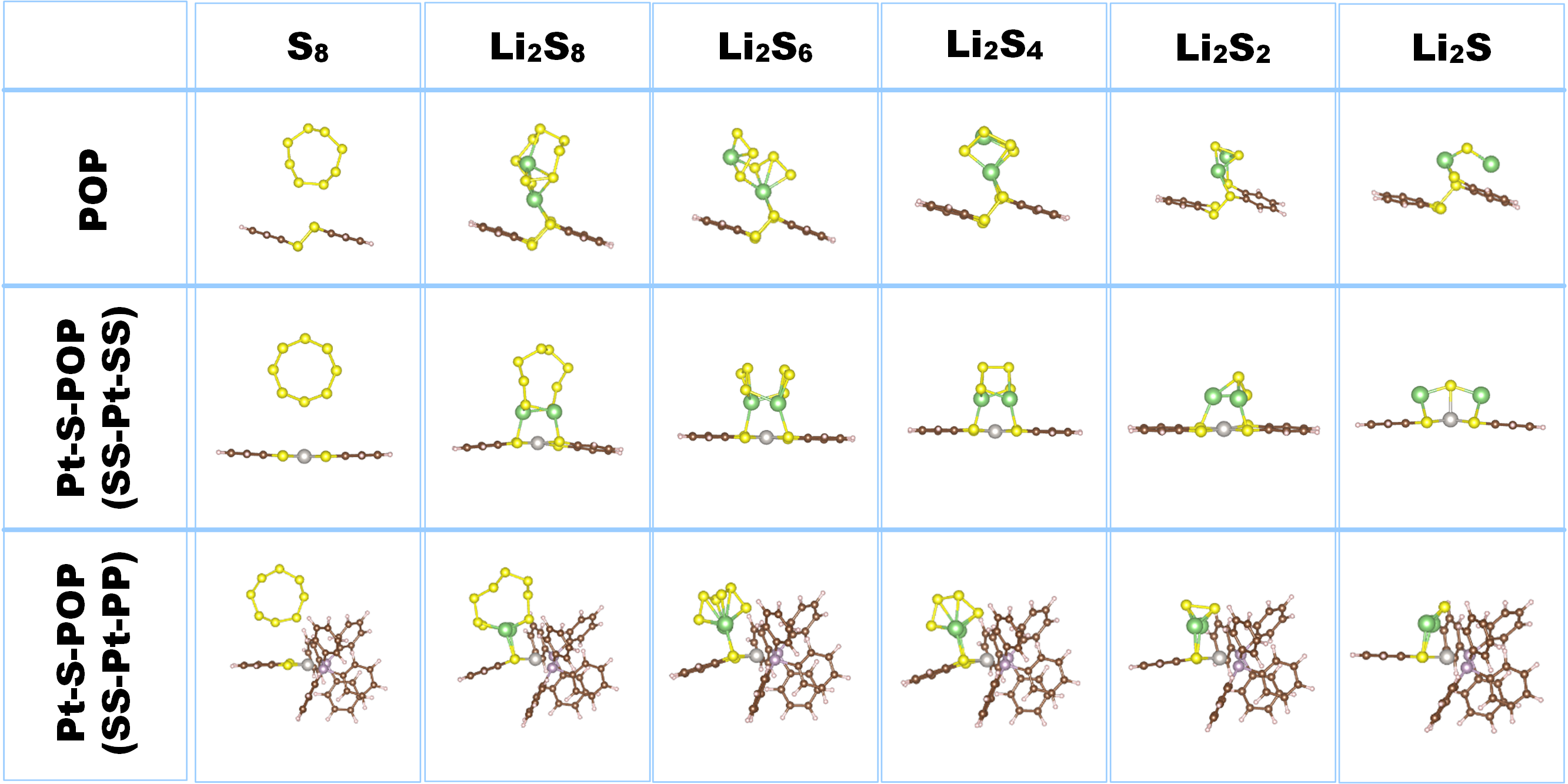


**Figure S30**. Optimized adsorption configurations for various LiPSs on the S-POP, Pt-S-POP (SS-Pt-SS, type I), and Pt-S-POP (SS-Pt-PP, type II).


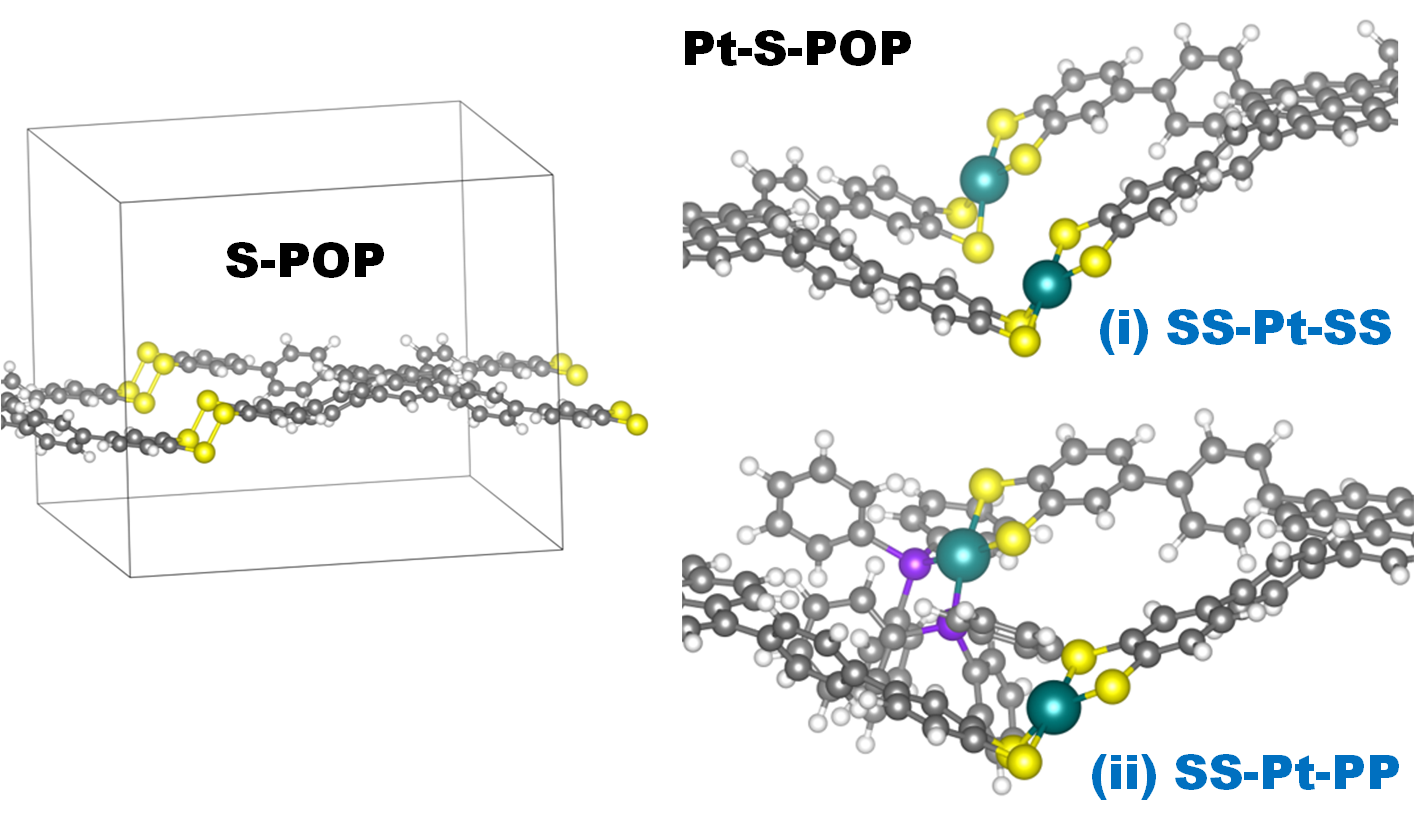


**Figure S31**. Simulated deformation charge density of S-POP, Pt-S-POP (SS-Pt-SS, type I), and Pt-S-POP (SS-Pt-PP, type II).

**Reference**

[S1] J. Zhang, G. Xu, Q. Zhang, X. Li, Y. Yang, L. Yang, J. Huang, G. Zhou, *Advanced Science* **2022**, 9, 2201579.

[S2] M. Zhou, Y. Li, T. Lei, W. Chen, G. Rao, L. Xue, A. Hu, Y. Fan, J. Huang, Y. Hu, X. Wang, J. Xiong, *Small* **2021**, 17, 2104367.

[S3] P. Guo, K. Sun, X. Shang, D. Liu, Y. Wang, Q. Liu, Y. Fu, D. He, *Small* **2019**, 15, 1902363.

[S4] P. Cheng, P. Guo, K. Sun, Y. Zhao, D. Liu, D. He, *Journal of Membrane Science* **2021**, 619, 118780.

[S5] Z. Cheng, Y. Chen, Y. Yang, L. Zhang, H. Pan, X. Fan, S. Xiang, Z. Zhang, *Advanced Energy Materials* **2021**, 11, 2003718.

[S6] B. Fan, Q. He, Q. Wei, W. Liu, B. Zhou, Y. Zou, *Carbon* **2023**, 214, 118361.

[S7] W. Jing, J. Zu, K. Zou, X. Dai, Y. Song, J. Han, J. Sun, Q. Tan, Y. Chen, Y. Liu, *Journal of Materials Chemistry A* **2022**, 10, 4833.

[S8] F. Ma, B. Yu, X. Zhang, Z. Zhang, K. Srinivas, X. Wang, D. Liu, B. Wang, W. Zhang, Q. Wu, Y. Chen, *Chemical Engineering Journal* **2022**, 431, 133439.

[S9] X. Qi, L. Huang, Y. Luo, Q. Chen, Y. Chen, *Journal of Colloid and Interface Science* **2022**, 628, 896.

[S10] L. Tan, X. Li, Z. Wang, H. Guo, J. Wang, *ACS Appl Mater Interfaces* **2018**, 10, 3707.

[S11] Q. Ma, M. Hu, Y. Yuan, Y. Pan, M. Chen, Y. Zhang, D. Long, *J Colloid Interface Sci* **2020**, 566, 11.

[S12] C. Shen, Y. Li, M. Gong, C. Zhou, Q. An, X. Xu, L. Mai, *ACS Applied Materials & Interfaces* **2021**, 13, 60046.

[S13] H. Zhang, Q. Liu, S. Ruan, C. Ma, X. Jia, W. Qiao, L. Ling, J. Wang, *Applied Surface Science* **2022**, 578, 152022.

[S14] W. Jing, K. Zou, X. Dai, M. Shi, J. Sun, D. Zhu, S. Guo, Y. Chen, Y. Liu, *Journal of Colloid and Interface Science* **2021**, 601, 305.

[S15] P. Wang, B. Xi, Z. Zhang, M. Huang, J. Feng, S. Xiong, *Angewandte Chemie International Edition* **2021**, 60, 15563.

[S16] Y. Tian, G. Li, Y. Zhang, D. Luo, X. Wang, Y. Zhao, H. Liu, P. Ji, X. Du, J. Li, Z. Chen, *Advanced Materials* **2020**, 32, 1904876.

[S17] A. E. Shrshr, Y. Dong, M. A. Al-Tahan, X. Kang, H. Guan, X. Zheng, J. Zhang, *Journal of Alloys and Compounds* **2022**, 910, 164917.

[S18] T. Xiao, Q. Chen, W. Zhong, M. Yang, F. Cai, W. Liu, M. Ren, Y. Wang, *Journal of Alloys and Compounds* **2022**, 907.

[S19] Z. Liu, Z. Hu, X. Jiang, Y. Zhang, X. Wang, S. Zhang, *Electrochim Acta* **2022**, 422, 140496.

[S20] Z. Yu, B. Wang, X. Liao, K. Zhao, Z. Yang, F. Xia, C. Sun, Z. Wang, C. Fan, J. Zhang, Y. Wang, *Advanced Energy Materials* **2020**, 10, 2000907.

[S21] Y. Zuo, Y. Zhu, Q. Wang, K. Lv, W. Su, Y. Tang, Y. Chen, *Journal of Materials Chemistry A* **2020**, 8, 20111.
